# Rapid building block-economic synthesis of long, multi-*O*-GalNAcylated MUC5AC tandem repeat peptides†

**DOI:** 10.1039/d3sc05006h

**Published:** 2023-12-18

**Authors:** Arseniy Galashov, Ekaterina Kazakova, Christian E. Stieger, Christian P. R. Hackenberger, Oliver Seitz

**Affiliations:** a Department of Chemistry, Humboldt-Universität zu Berlin Brook-Taylor-Straße 2 12489 Berlin Germany oliver.seitz@chemie.hu-berlin.de; b Leibniz-Forschungsinstitut für Molekulare Pharmakologie (FMP) Robert-Rössle-Strasse 10 13125 Berlin Germany

## Abstract

The study of mucin function requires access to highly *O*-glycosylated peptides with multiple tandem repeats. Solid-phase synthesis would be a suitable method, however, the central problem in the synthesis of mucin glycopeptides is the need to use precious and potentially vulnerable glycoamino acid building blocks in excess. In this article, we report the development of a method based on SPPS and native chemical ligation/desulfurization chemistry that allows the rapid, reliable, and glyco-economical synthesis of long multi-*O*-GalNAcylated peptides. To facilitate access to the glycosyl donor required for the preparation of Fmoc-Ser/Thr(αAc_3_GalNAc)-OH we used an easily scalable azidophenylselenylation of galactal instead of azidonitration. The problem of low yield when coupling glycoamino acids in small excess was solved by carrying out the reactions in 2-MeTHF instead of DMF and using DIC/Oxyma. Remarkably, quantitative coupling was achieved within 10 minutes using only 1.5 equivalents of glycoamino acid. The method does not require (microwave) heating, thus avoiding side reactions such as acetyl transfer to the N-terminal amino acid. This method also improved the difficult coupling of glycoamino acid to the hydrazine-resin and furnished peptides carrying 10 GalNAc units in high purities (>95%) of crude products. Combined with a one-pot method involving native chemical ligation at a glycoamino acid junction and superfast desulfurization, the method yielded highly pure MUC5AC glycopeptides comprising 10 octapeptide tandem repeats with 20 α-*O*-linked GalNAc residues within a week.

## Abstract 2

New methods allow the very smooth synthesis of highly pure MUC5AC glycopeptides containing 20 GalNAc units on 10 tandem repeats within a week.

## Graphical abstract

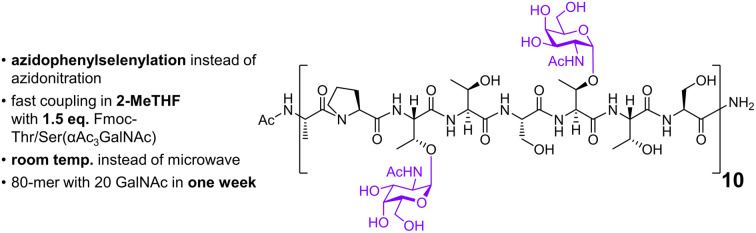

## Introduction

**Fig. 1:**
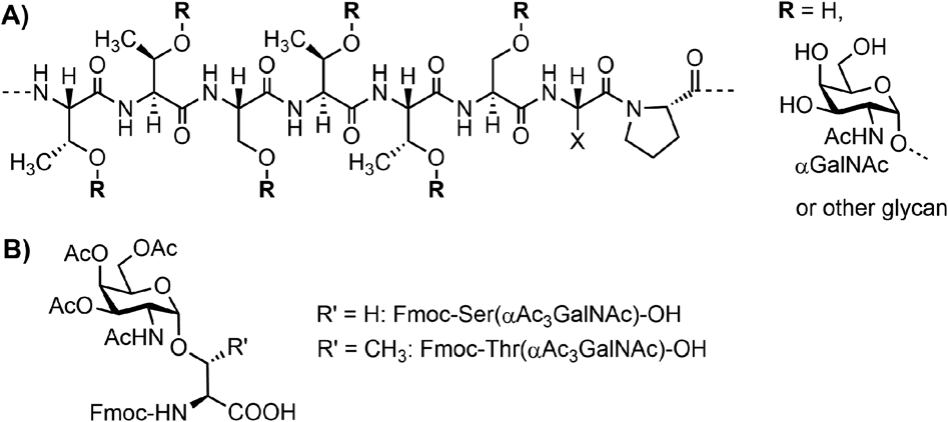
(A) Tandem repeat motif (TTSTTSXP) for MUC5AC. (B) Fmoc-Ser/Thr(αAc_3_GalNAc)-OH building blocks.

Mucins are the major constituent of the mucus layer that protects the epithelium from direct contact with the external environment.^1–3^ A characteristic hallmark of mucins is the presence of highly *O*-glycosylated multiple tandem repeats (TRs). For example, MUC5AC contains approximately 240 copies of a TTSTTSXP motif, with Thr and Ala being the most frequent amino acids in position X (Fig. 1A).^4^ Each Ser or Thr residue can occur in *O*-glycosylated form. In diseases, glycosylation patterns and the number of TRs can change dramatically.^5^ For example, malignancies are typically accompanied by increased occurrence of the Tn (αGalNAc), T (Galβ1-3GalNAcα1) and sialyl-Tn antigen structures.^6,7^ Therefore, this region is of the highest interest to researchers looking for ways to decipher the functional consequences.

Due to difficulties with the cloning of extended repeated sequences and the microheterogeneity of native glycans, conventional methods based on genetic engineering cannot provide access to mucin TR regions of defined length and glycan composition. Instead, chemical synthesis has been used to provide glyco-defined mucins, most frequently for applications in immunological research.^8–10^ Many research groups have contributed to the advancement of the synthesis methodology.^11^ While N-linked glycopeptides can be prepared by connecting elaborated carbohydrates with the full-length peptide, no such approach is feasible for the synthesis of long mucin peptides. Instead, the synthesis of *O*-glycopeptides relies on preformed glycoamino acids, with Fmoc-Ser/Thr(αAc_3_GalNAc) as the most frequently used building blocks (Fig. 1B).

Synthesis of *O*-glycopeptides is challenging. Couplings to and with glycoamino acids are more difficult than with conventional amino acid building blocks. For one thing, reactivity is low due to steric requirements. For another, glycoamino acids run the risk of racemization or β-elimination reactions under forced coupling conditions.^12,13^

Most of the previous work yielded *O*-glycopeptides that rarely contained more than 20 amino acids and only a few glycan units, some of which were of impressive complexity.^14–25^ However, the inherent properties of mucins, such as viscoelasticity, gelation, shielding, and multivalency, emerge from multirepeat TRs.^26–28^ We, therefore, embarked on a research program focusing on the development of methods for the synthesis of multi TR mucins. In the synthesis of long mucin peptides containing many GalNAc units, the difficulties of *O*-glycopeptide synthesis become even more apparent. A typical approach to get around difficult steps in solid-phase peptide synthesis is to use the building blocks in large excess and employ double couplings. Even with commercially available Fmoc-Ser/Thr(αAc_3_GalNAc), the easiest glycoamino acids to synthesize, such an approach is extremely costly if a large number of glycosylated building blocks must be incorporated. Notable contributions to the synthesis of highly GalNAcylated MUC1 and MUC2 TRs, with up to 141 amino acids in length, have been reported.^29–32^ Middle-sized segments (<40 amino acids) containing up to 7 GalNAc units were synthesized on solid phase either by single amino acid extension or fragment condensation. Purified fragments were subsequently stitched together by a series of silver-assisted thioester ligations^29^ or Cu-click reactions.^30^ Recently, Wang^31^ described three native chemical ligation (NCL) reactions used to convergently assemble a 240 amino acid antifreeze protein from two 30-mers containing none or 10 GalNAc residues. A common theme of the solid-phase synthesis tactics used to access fragments for ligation is that Fmoc-Thr(αAc_3_GalNAc) building blocks were coupled by using a twofold excess.^30–32^ Considering the reported difficulties,^12^ this number of excess equivalents is small. Possible truncations, side reactions, and purity of the products prior to purification have not been reported.

Herein, we describe the development of an efficient and economically viable method for the smooth synthesis of highly *O*-GalNAcylated mucin multi tandem repeats. We report an optimized multigram synthesis of both Fmoc-Thr(αAc_3_GalNAc) and Fmoc-Ser(αAc_3_GalNAc) based on azidophenylselenylation of galactal.^33^ During the optimization of glycoamino acid coupling, we observed acetyl transfer reactions resulting in the undesired capping of the growing peptide chain. Most surprising were the results from a thorough analysis of coupling conditions, which led to a method enabling quantitative couplings within 10 minutes by using only 1.5 equivalents of glycoamino acid. This method also facilitated the notoriously unreliable loading of the hydrazine resin required for the synthesis of peptide thioesters according to the methods of Liu and Dawson.^34,35^ Combined with an extremely efficient ligation–desulfurization sequence the new method enabled the smooth synthesis of 10 TR long MUC5AC peptides containing 20 GalNAc residues on Ser or Thr.

## Results and discussion

### General considerations

The arguably most robust access to long peptides is provided by native chemical ligation.^36–39^ MUC5AC contains multiple copies of a Thr- and Ser-rich octapeptide TR, with TTSTTSXP as a typical representative.^4^ Considering the absence of cysteine and the occurrence of Ala as a frequently occurring amino acid at position X, we targeted a Ser-Cys junction, which can be converted to the native Ser-Ala junction by desulfurization. In our retrosynthetic analysis, we planned the solid-phase synthesis of 40-mers comprising GalNAc residues at 25% of the amino acids. Of particular interest for us was to explore NCL with thioesters containing glycoamino acid at the C-terminus. The resulting need for substantial amounts of Fmoc-Thr/Ser(αAc_3_GalNAc) building blocks prompted us to develop both a reliable and affordable pipeline for their preparation and their economic use in peptide chemistry.

### Multigram-scale preparation of glycoamino acids

A range of different approaches to the synthesis of Tn antigen has been described in the literature.^40–46^ Usually, peracetylated galactal serves as a precursor for a 2-azido-2-deoxygalactosyl donor, which is required for achieving α-selectivity in the glycosylation of serine or threonine. The introduction of the azido group is commonly performed by azidonitration with ceric ammonium nitrate. This reaction yields an anomeric nitrate, which is converted to galactosyl acetate or halide. Azidonitration of galactal is cumbersome, often low yielding, and as Ratcliffe and Lemieux stated: “it must be realized that hazardous conditions may be encountered.”^47^ In our labs, azidonitration was a bottleneck, and we therefore opted to explore alternative chemistries. The Nifantiev group reported a safe, reliable, and high-yielding homogeneous azidophenylselenylation of glycals.^33^ The formed selenoglycosides have been used for 1,2-*cis*-selective glycosylation reactions.^41,48^ Although it is difficult to compare the combined toxicity of ceric ammonium nitrate and sodium azide used in azidonitration with the anticipated toxicity of organic selenides, avoidance of sodium azide in azidophenylselenylation reduces the risk of explosion in large scale synthesis.

**Scheme 1:**
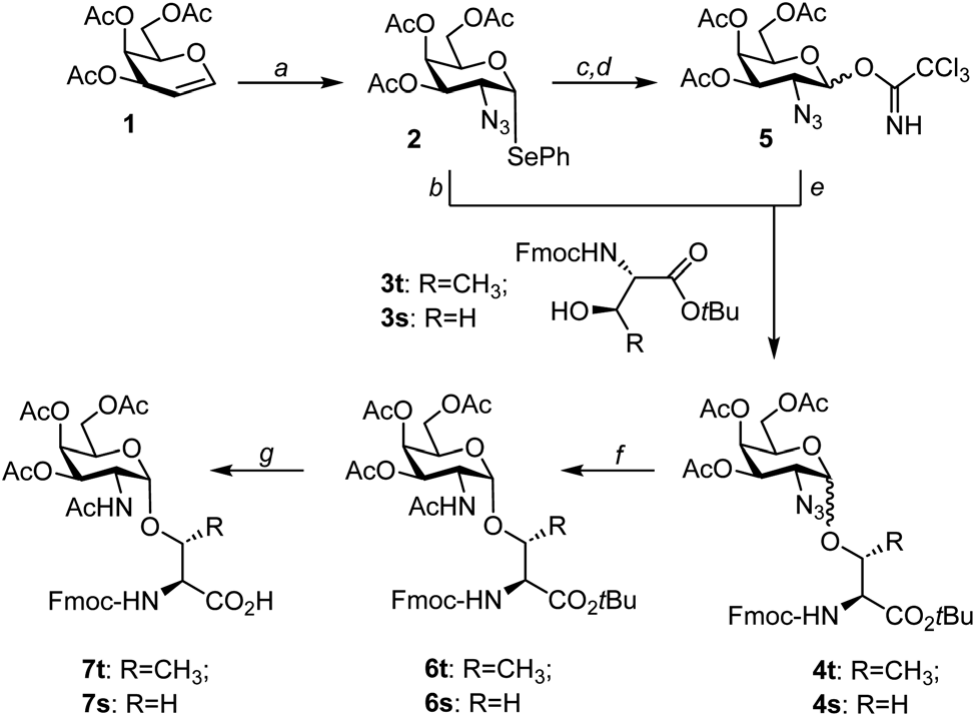
Synthesis of Fmoc-Thr(αAc_3_GalNAc)-OH and Fmoc-Ser(αAc_3_GalNAc)-OH. Conditions: (a) Se_2_Ph_2_, trimethylsilyl azide, PhI(OAc)_2_, CH_2_Cl_2_, −30 °C (55%); (b) *N*-iodosuccinimide, trimethylsilyl triflate, CH_2_Cl_2_, Et_2_O, 0 °C; (c) *N*-iodosuccinimide, acetone, H_2_O, RT (quant.); (d) CCl_3_CN, Cs_2_CO_3_, CH_2_Cl_2_, RT (84%); (e) trimethylsilyl triflate, CH_2_Cl_2_, Et_2_O, 0 °C; (f) Zn, THF, AcOH, Ac_2_O, RT; (g) TFA, CH_2_Cl_2_, RT (70–75%).

For the purpose of an application in the α-selective glycosylation of Fmoc-threonine *tert*-butyl ester 3t, 2-azido-2-deoxy-phenylselenogalactosyl donor 2 was prepared from triacetylgalactal 1 as previously described (Scheme 1).^33,48^ Next, this donor was allowed to react with the threonine aglycon at −10 °C under activation with NIS and TMSOTf in a mixture of diethyl ether and dichloromethane. The 2-azido-galactosyl-threonine conjugate 4t was formed as a 2.5 : 1 mixture of α- and β-isomers (Table 1). Chromatographic purification proved challenging. Therefore, the α/β-mixture was subjected to the zinc-mediated azide reduction in the presence of acetic anhydride furnishing the *N*-acetylgalactosamine-threonine conjugate 6t, which was easier to purify than 4t. After chromatography, the α-isomer 6t was isolated in 24% yield. The same synthetic route was applied to the glycosylation of Fmoc-serine *tert*-butyl ester 3s, providing the α-linked serine conjugate 6s in 26% yield.

**Table 1:** Approaches to the glycosylation of Fmoc-Thr(OH)-O^*t*^Bu and Fmoc-Ser(OH)-O^*t*^Bu

Donor	Acceptor	α : β ratio after glycosylation^a^	Yield for two steps (glycosylation + azide reduction),^b^ %
2	3t	2.5 : 1	24
2	3s	2.7 : 1	26
5	3t	2.3 : 1	47^c^
5	3	2.7 : 1	51^c^

a Ratio was determined by NMR of crude.

b Only for α-isomer.

c Gram-scale reactions yields are shown.

The relatively moderate yields of the glycosylation, caused by difficult separation from by-products, prompted us to consider an alternative donor. The 2-azido-2-deoxygalactosyl trichloroacetimidate 5 was readily prepared from selenogalactoside 2 in 84% yield in two steps. First, 2 was quantitatively hydrolyzed to hemiacetal S1 (Scheme S1†), followed by the known reaction with trichloroacetonitrile.^44,49^ Glycosylation of amino acid 3t was induced upon the addition of trimethylsilyl triflate. With a 2.3 : 1 ratio of α/β-anomers, this reaction provided comparatively the same α-selectivity as the glycosylation with the selenophenyl donor 2 but almost twice as high yields (Table 1). Importantly, the outcome was independent of the reaction scale (100 mg to 9.3 g). To facilitate the purification of 6 after reductive acetylation, it proved beneficial to adjust the donor/acceptor ratios. A 1 : 1.1 ratio allowed the isolation of Fmoc-Thr(αAc_3_GalNAc)-O^*t*^Bu in 47% yield over two steps. For the synthesis of Fmoc-Ser(αAc_3_GalNAc)-O^*t*^Bu, the donor/acceptor ratio was adjusted to 1.2 : 1. The α-selectivity and the yields were slightly higher than for glycosylation of the threonine derivative. Cleavage of the *tert*-butyl ester was uneventful and furnished the glycoamino acid building blocks 7t and 7s on a multigram scale.

### Loading of (glyco)amino acid to the NH_2_-NH-TRT resin

The synthesis of glycopeptide thioesters used in NCL chemistry is most easily carried out on hydrazine resins.^50,51^ The glycopeptide hydrazides obtained after TFA cleavage are converted to glycopeptide thioesters under mildly acidic conditions.^35^ The retrosynthetic analysis called for a loading of the H_2_N–NH–trityl resin with Fmoc-Ser(O^*t*^Bu)-OH or Fmoc-Ser(αAc_3_GalNAc)-OH. In our laboratories, loading this resin led to notoriously inconsistent results. While problematic couplings to N-terminal azapeptides have been described in a recent review,^52^ difficulties with the H_2_N–NH–trityl resin have apparently not been reported. Usually, loading is performed with HATU/DiPEA, although similar methods have been reported with HBTU,^53^ HCTU,^54^ and PyAOP.^55^

To evaluate various coupling conditions, we prepared a Tentagel R Fmoc-NH-NH-TRT resin from Tentagel R Cl-TRT resin.^56^ This low loading resin was used because of its known advantages in the synthesis of long and difficult peptides. However, instead of suspending Fmoc-NH-NH_2_ in DMF/CH_2_Cl_2_, we used anhydrous DMSO as a solvent. The Fmoc-load was determined before Fmoc cleavage and after subsequent coupling of the starting amino acid. Conventional double coupling of Fmoc-Ser(O^*t*^Bu)-OH (10 eq.) under activation with HATU in the presence of DiPEA (condition 1, Table 2) afforded only 49% loading yield. Microwave heating did not remedy the situation (condition 2). To avoid the possible formation of guanidino adducts, PyOxim was used as an activator with little success (condition 3). A higher loading yield was achieved when a coupling with DIC/Oxyma activation was performed overnight (condition 4). In our further attempts, we were inspired by the work of de la Torre/Albericcio^57^ and the Schönleber/Pedersen groups,^58^ who used 2-MeTHF as a solvent for coupling reactions promoted by DIC/Oxyma. Replacing DMF with 2-MeTHF dramatically improved reaction rates (conditions 5 and 6). Quantitative yields were obtained after 10 min reaction time.

**Table 2:** Coupling systems for loading of Fmoc-Ser(O^*t*^Bu)-OH to the NH_2_-NH-TRT resin

Coupling conditions^a^	Yield^b^ (%)
(1) 2 × HATU/DiPEA, 45 min in DMF, rt	49
(2) 2 × HATU/DiPEA, 30 min each in DMF, 50 °C	26
(3) 3 × PyOxym/DiPEA, 5 min in DMF, 75 °C	25
(4) 1 × DIC/Oxyma/DiPEA, 17 h in DMF, rt	63
(5) 1 × DIC/Oxyma, 5 min in 2-MeTHF, rt	92
(6) 1 × DIC/Oxyma, 10 min in 2-MeTHF, rt	100

a 10 eq. of amino acid and activating reagents were used, 20 eq. of DiPEA was used. In condition 4 1 eq. of DiPEA was added.

b Yields calculated according to Fmoc-test. In conditions with 2-MeTHF preactivation (2 min) was used.

Next, we investigated the non-trivial loading of the H_2_N–NH–TRT resin with Fmoc-Ser(αAc_3_GalNAc)-OH (Table 3). Given its high cost, coupling with 10 equivalents is not an option. Gratifyingly, with the DIC/Oxyma system in 2-MeTHF, a 75% loading yield was obtained by using only 1 eq. glycoamino acid (condition 1, Table 3). Increasing the coupling time from 2 to 20 hours did not improve the yield (condition 2), but with only 0.5 excess equivalents, >90% yield was achieved (condition 3), which was deemed sufficient in terms of yield and building block economy.

**Table 3:** Optimizing loading of NH_2_–NH–TRT resin with Fmoc-Ser(αAc_3_GalNAc)-OH (7s)^a^

Coupling conditions	Yield^b^ (%)
(1) 1 eq. 7s/DIC/Oxyma, 2 h in 2-MeTHF, rt	75
(2) 1 eq. 7s/DIC, 10 eq. Oxyma, 20 h in 2-MeTHF, rt	71
(3) 1.5 eq. 7s/DIC, 15 eq. Oxyma, 2 h in 2-MeTHF, rt	92

a AA = Fmoc-Ser(αAc_3_GalNAc)-OH.

b Yields calculated according to Fmoc-test.

### Optimizing coupling of glycoamino acid to peptide chain

**Scheme 2:**
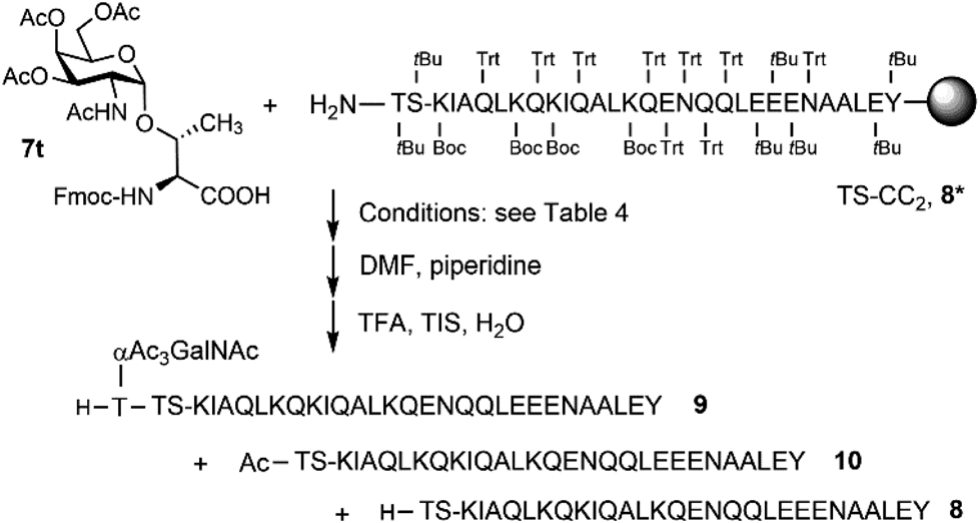
Optimizing coupling of Fmoc-Thr(αAc_3_GalNAc)-OH (7t).

**Fig. 2:**
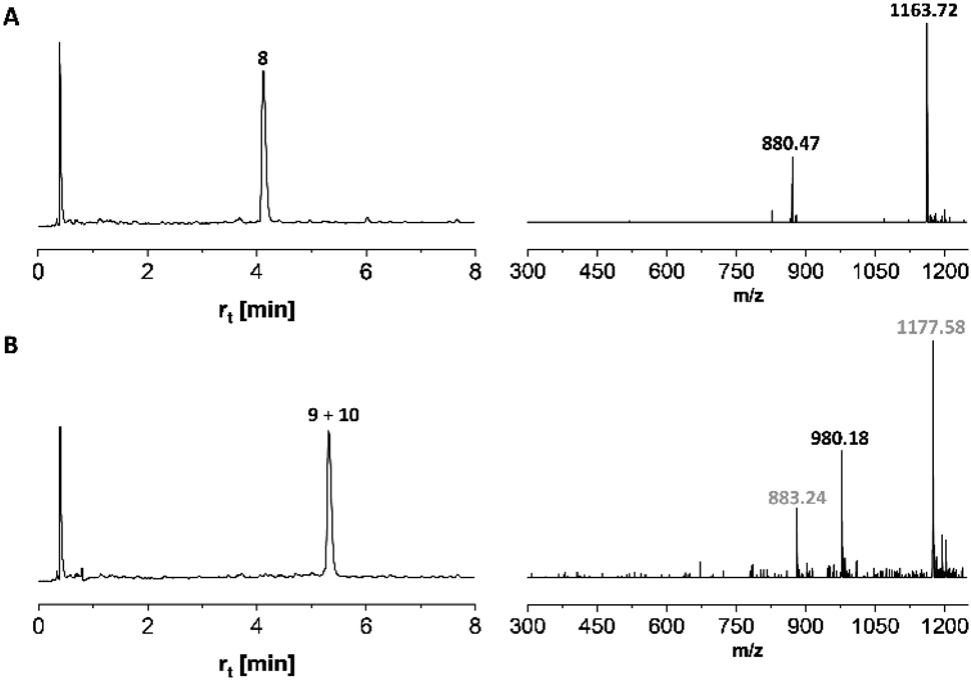
UPLC and ESI-MS analysis of (A) peptide 8 cleaved from 8* before and (B) after coupling of glycoamino acid 7t. Gray numbers correspond to mass peaks of peptide 10. Gradient 18–30% ACN in water + 0.1% TFA. *λ* = 210 nm.

Microwave-assisted SPPS is a powerful tool that can facilitate the synthesis of aggregating peptides and dramatically improve the yield of synthesized peptide while reducing the time required for synthesis.^59^ It has been reported that the method has the potential to improve the synthesis of *O*-glycosylated mucin and antifreeze peptides.^60–62^ Therefore, we investigated the coupling of Fmoc-Thr(αAc_3_GalNAc)-OH onto a resin-bound peptide under microwave conditions. Due to our interest in using coiled coil interactions for the assembly of large glycopeptide structures, we explored the extension of a slightly modified coiled coil peptide^63^ TS-CC_2_, 8* (Scheme 2). This peptide was prepared according to a recently described method^64^ involving Fmoc-deprotection for 1 minute at 90 °C with 20% piperidine in DMF and coupling of amino acids with DIC/Oxyma for 2 minutes at 90 °C. HPLC-MS analysis of the crude material obtained upon TFA cleavage (Fig. 2A) confirmed the efficiency of this synthesis protocol, which provided the 30-mer peptide in very high purity. For coupling of 7t, we extended the coupling time to 5 min (condition 1, Table 4). Spectrometric monitoring of Fmoc loads suggested quantitative coupling yields. Furthermore, the HPLC trace of the crude material from the TFA cleavage indicated smooth coupling, as inferred from peak-to-peak conversion (Fig. 2B). However, MS analysis showed that the extension of 8* with Fmoc-Thr(αAc_3_GalNAc)-OH led to the formation of two products. The *m*/*z*-ratios correspond to the desired extension product 9 and a truncation product 10 obtained by acetylation of 8*. Since no capping was used, the acetyl group must be derived from the glycoamino acid. Aminolysis of acetic acid esters by the N-terminal amino group is probably facilitated by microwave heating. This and the difficulty in separating the two products prompted us to explore alternative coupling conditions.

**Table 4:** Optimizing the coupling of Fmoc-Thr(αAc_3_GalNAc)-OH (7t) acid to the resin-bound peptide 8* in DMF

Coupling conditions	Yield^a^ (%)
(1) 1.5 eq. 7t/DIC/Oxyma, 5 min, 75 °C	NA
(2) 1.5 eq. 7t/HATU/HOAt, 3 eq. DiPEA, 30 min, rt	75
(3) 1.5 eq. 7t/HATU/HOAt, 3 eq. DiPEA, 60 min, rt	78
(4) 1.5 eq. 7t/HATU/HOAt, 3 eq. TMP, 30 min, rt	70
(5) 1.5 eq. 7t/HATU/HOAt, 3 eq. TMP, 60 min, rt	82
(6) 1.5 eq. 7t/HATU/HOAt, 3 eq. TMP, 90 min, rt	82
(7) 1.5 eq. 7t/HATU/HOAt, 3 eq. TMP, 30 min, rt	82
(8) 1.5 eq. 7t/HATU/HOAt, 3 eq. TMP, 60 min, rt	90
(9) 1.5 eq. 7t/HATU/HOAt, 3 eq. TMP, 90 min, rt	92
(10) 2.25 eq. 7t/HATU/HOAt, 4.5 eq. TMP, 90 min, rt	94
(11) 2 × [0.95 eq. 7t/HATU/HOAt, 1.9 eq. TMP, 30 min, 40 °C]	89
(12) 2 × [1.15 eq. 7t/HATU/HOAt, 2.3 eq. TMP, 30 min, 40 °C]	93
(13) 2 × [1.3 eq. 7t/HATU/HOAt, 2.6 eq. TMP, 30 min, 40 °C]	97
(14) 2 × [1.5 eq. 7t/HATU/HOAt, 3 eq. TMP, 18 min, rt]	100

a Yields calculated by integrating peaks in UPLC traces. Based on four independent replicates performed for condition 10, a 4% standard deviation is estimated. TMP, 2,4,6-trimethylpiperidine.

In search of (glyco) efficient coupling, we reviewed conditions based on the use of HATU as one of the strongest activation reagents.^65^ We used DMF as a solvent and varied the base, reaction time, and temperature (Table 4). Again, couplings were carried out at the resin-bound 30-mer peptide 8*, and again, rather than simply determining the Fmoc load, we monitored the reactions by HPLC analysis of crudes obtained after TFA cleavage (Fig. S5†). With only 1.5 equivalents of glycoamino acid/HATU/HOAt, the reaction required 90 minutes to afford a 92% yield (conditions 9). Based on a report from the Gildersleeve lab on the epimerization of glycoamino acids with DIPEA^12^ and slightly slower couplings (compare conditions 2 and 3 with 4 and 5), we focused on the use of TMP as a base. Increasing the excess from 1.5 to 2.25 equivalents at a 90 minute reaction time provided only a minor improvement in coupling yields (conditions 10). With an aim to reduce coupling time, we investigated mild microwave irradiation (25 W) and increased the temperature to 40 °C (conditions 11–13). At the same time, double coupling (18–30 min each) was performed with a total of 1.9–3 equivalents. At this temperature, we did not observe acetyl transfer. A double coupling for 18 min with 1.5 equivalents each provided a quantitative yield (condition 14). However, the need for 3 equivalents in each glycoamino acid coupling is costly.

Motivated by success with difficult couplings to the hydrazine resin, we explored reactions in 2-MeTHF with activation by the DIC/Oxyma system (Table 5). In our first attempt, a double coupling with substoichiometric amounts of glycoamino acid was carried out with little success. Remarkably, a single coupling with 1.5 equivalents of glycoamino acid in 2-MeTHF for 10 min proved sufficient to give a quantitative yield. No heating was required, and there was no evidence of acetyl transfer (Fig. S6†).

**Table 5:** Coupling of Fmoc-Thr(αAc_3_GalNAc)-OH (7t) in 2-MeTHF to peptide 8*

Coupling conditions	Yield^a^ (%)
(1) 2 × [0.78 eq. 7t/DIC, 10 eq. Oxyma in 2-Me-THF, 18 min, rt]	56
(2) 1.09 eq. 7t/DIC/Oxyma in 2-MeTHF, 10 min, rt	94
(3) 1.5 eq. 7t/DIC/Oxyma in 2-MeTHF, 10 min, rt	100

a Yields calculated by integrating peaks in UPLC chromatograms. For condition 1 double coupling was performed.

The highly efficient coupling of Fmoc-Ser/Thr(αAc_3_GalNAc) in 2-Me-THF is noteworthy. Research by de la Torre and Albericcio has shown that coupling reactions with DIC/Oxyma activation proceed remarkably swiftly in 2-MeTHF, whereas DMF seemed to be a better solvent for activation with HATU or HBTU.^57^ There is an increasing amount of evidence to suggest that the rate of coupling reactions increases with decreased polarity of the solvent.^58,66^ We assume that the high reactivity in apolar solvents can counterbalance the reduction of reaction rate caused by low concentration of the building block. Hence, apolar solvents such as 2-MeTHF could prove particularly advantageous when expenses for building blocks are high.

### Solid-phase synthesis of glycopeptides

**Scheme 3:**
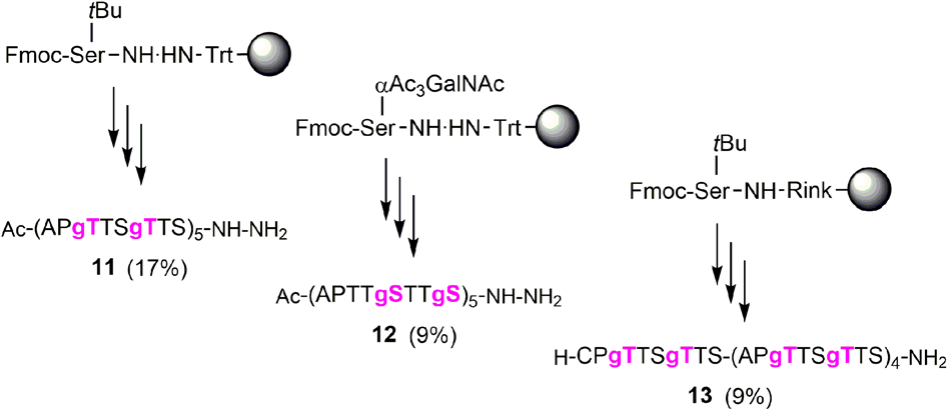
Solid-phase synthesis of MUC5AC glycopeptides and their yields after isolation in parentheses. gS, gT = Ser/Thr(αGalNAc). Conditions: Fmoc-cleavage: 20% piperidine in DMF; coupling: HATU/Oxyma/DiPEA (5 eq., 5 eq., 15 eq.); coupling of 7t or 7s: see condition 3, Table 5; capping: Ac_2_O/DiPEA/DMF (70 : 20 : 10, v/v/v); TFA cleavage: for 11 and 12 TFA/TIS/H_2_O (96/2/2). For 13 TFA/TIS/EDT/H_2_O (94/2/2/2).

**Fig. 3:**
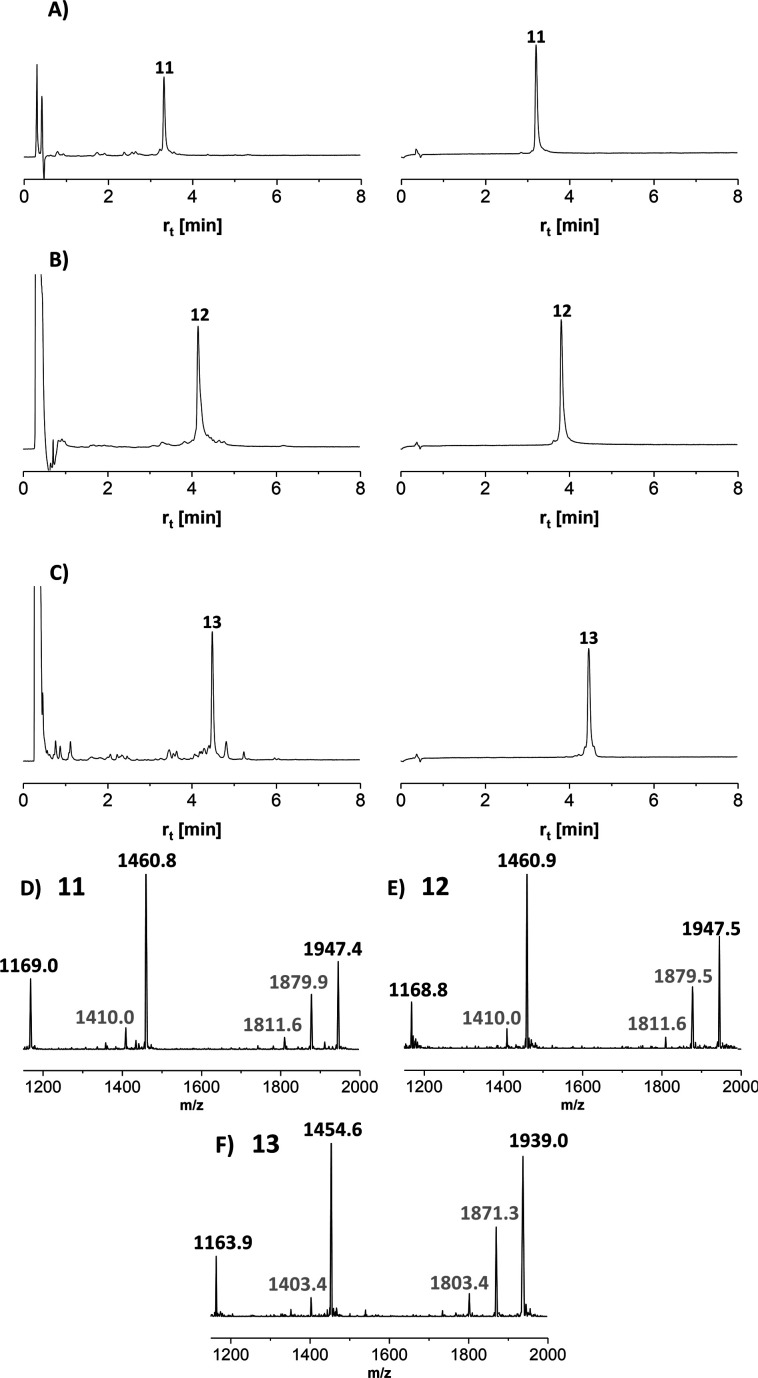
UPLC analysis of glycopeptides (A) 11, (B) 12, and (C) 13 before (left) and after (right) HPLC purification. Gradient: 07–17% ACN in water + 0.1% TFA. *λ* = 210 nm. ESI-MS spectra of glycopeptides (D) 11, (E) 12 and (F) 13. Gray numbers indicate mass peaks due to fragmentation during ESI-MS measurement.

Next, we embarked on the solid phase synthesis of 5 TRs long MUC5AC peptides that can be used in NCL chemistry (Scheme 3). Couplings of the glycoamino acids were performed in 2-MeTHF with 1.5 eq. of glycoamino acid, 1.5 eq. of DIC, and 1.5 eq. of Oxyma. The other amino acids were introduced in DMF solutions by using 5 eq. of amino acids and activation with HATU/Oxyma in the presence of DiPEA. In test cleavages of glycopeptides, we observed that *O*-acetyl-protected glycopeptides are unstable in aqueous acidic solution. The glycan residues lose acetyl protecting groups, resulting in complicated HPLC profiles (Fig. S7†). If necessary, this side reaction can be suppressed by dissolving the glycopeptides in buffer solutions at pH 7. Preferably, however, the acetyl protecting groups were removed prior to cleavage by treating the resin with a N_2_H_4_·H_2_O/DMF mixture. Under these conditions, the synthesis of the 40-mer peptide hydrazides 11 and 12, containing 10 glycosylated amino acids, proceeded remarkably smoothly, as evidenced by the high purity of crude material (Fig. 3A and B). The situation was somewhat different for the synthesis of glycopeptide 13, which contains an N-terminal cysteine for NCL. Here, the crude material showed a few by-products that were removed by preparative HPLC (Fig. 3C).

MS analysis of the glycopeptides showed peaks with *m*/*z* values (−200 Da and −400 Da) corresponding to a loss of one or two GalNAc residues (Fig. 3D–F). This loss did not occur when glycopeptides were detached in *O*-acetylated form (Fig. S8†). Hydrolytic cleavage of sugar residues could occur during detachment with TFA. We therefore prepared glycopeptide 12 with *O*-acetyl groups on the GalNAc residues (which is known to increase their hydrolytic stability) and performed the *O*-deacetylation in solution. The mass spectra did not change (Fig. S9†). Therefore, we attributed the loss of GalNAc-residues to the conditions of the ESI-MS measurement. In the absence of readily ionizable side chains, protonation may occur also at the sugar, followed by possible cleavage of the glycosidic bond. Most importantly, this fragmentation reaction can be prevented by altering the conditions of ESI-MS (*vide infra*).

### One-pot native chemical ligation/desulfurization of glycopeptides

**Scheme 4:**
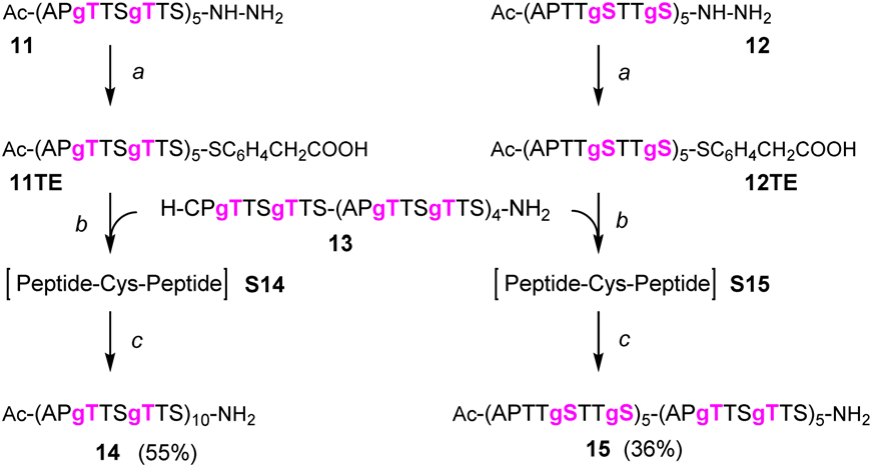
NCL/desulfurization affords MUC5AC peptides with 10 TRs and 20 GalNAc moieties. (a) 1.5 eq. acac, 200 mM MPAA, 6 M Gd-HCl, pH 3.1; (b) 2.5 mM peptides, 25 mM TCEP-HCl in 6 M Gd-HCl, 0.2 M Na_2_HPO_4_, pH 7.1; (c) EtOAc extraction, then: 0.8 mM peptide, 6 M Gd-HCl, 0.2 M sodium citrate, 200 mM TCEP-HCl, 100 mM NaBEt_4_, pH 4.5.

**Fig. 4:**
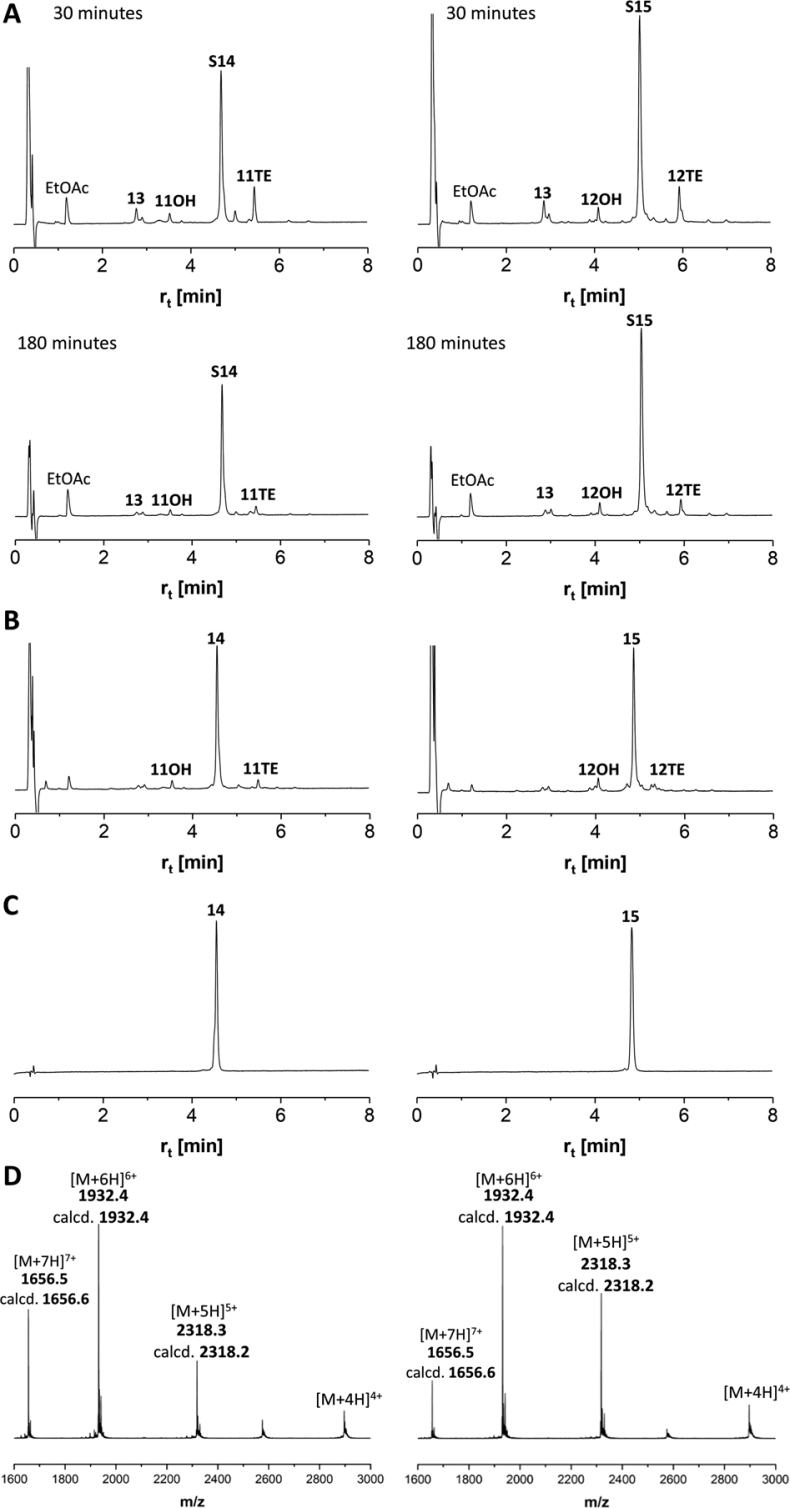
HPLC analysis of (A) NCL 13 + 11TE → S14 (left) or NCL 13 + 12TE → S15 (right), (B) desulfurization S14 → 14 (left) or S15 → 15 (right) and (C) purified products. 11OH and 12OH are hydrolysis products formed from corresponding thioesters. (D) ESI-HRMS analysis of purified MUC5AC peptides 14 (left) and 15 (right). *m*/*z* assignments for most abundant isotope.

We continued the synthesis with an NCL reaction between glycopeptides 11 and 13 (Scheme 4). First, 11 was converted to the peptide thioester using Dawson's Knorr-pyrazole method.^34^ After treatment with acetylacetone in the presence of mercaptophenylacetic acid (MPAA) for 90 minutes, the peptide hydrazide was quantitatively converted to a mixture of peptide-pyrazolate and peptide-MPAA thioester, and complete conversion to the peptide-MPAA thioester was observed after a total of 150 minutes (Fig. S10†). Without purification, glycopeptide 13 was added, and the buffer was adjusted to pH = 7.1. At the unhindered Ser-Cys junction, NCL was fast and afforded 95% ligation yield after only 30 min (Fig. 4A, left). We next examined the NCL between fragments 12 and 13. This reaction involves the formation of a Ser(GalNAc)–Cys junction, which cannot be avoided if GalNAcylation also extends to serine residues. We expected reactions at the more sterically demanding Ser(GalNAc) site to proceed slower than reactions at the C-terminal Ser. Contrarily, thioester formation at 12 proceeded faster than at 11, requiring only 60 min instead of 150 minutes for completion (Fig. S11†). Likewise, NCL with glycopeptide 13 proceeded smoothly, providing 95% ligation yield after only 30 minutes (Fig. 4A, right).

The mixtures of the NCL reactions were forwarded for desulfurization. We applied a very recent method from the Li group, which has been reported to enable almost instantaneous desulfurization upon reaction with tetraethylborate.^67^ A slight shift of retention to shorter times (compare Fig. 4A with 4B), consistent with the loss of a hydrophobic mercapto group, indicated that the reaction was completed after 1 minute. No side reactions were observed in the process of desulfurization. Of note, the apparent purity of crude products 14 and 15, both of which carry 20 GalNAc residues on 10 MUC5AC tandem repeats, was higher than 95%. After preparative HPLC and lyophilization, the glycopeptides 14 and 15 were obtained in 55% and 36% yield, respectively. To prevent fragmentation during high-resolution ESI-MS, it proved necessary to reduce the concentration of the collision gas (Fig. 4D).

## Conclusion

We developed a glyco-economic synthesis of long and highly *O*-GalNAcylated mucin peptides. The method relies on Fmoc-SPPS and NCL/desulfurization chemistry. Several bottlenecks had to be removed during method development.

(1) We introduced a facilitated, more reliable, and easily scalable synthesis of Fmoc-Thr/Ser(αAc_3_GalNAc)-OH, in which the potentially hazardous azidonitration of tri-*O*-acetylgalactal was replaced with azidophenylselenylation.

(2) For the challenging loading of hydrazine resin with a glycoamino acid coupling was performed in 2-MeTHF with DIC/Oxyma activation, which reliably provided higher yields than coupling in DMF.

(3) This approach also eliminated the third and probably most limiting bottleneck, namely the need for large amounts of expensive glycoamino acids in coupling reactions. By using 2-MeTHF as a solvent with DIC/Oxyma activation agents, only 1.5 equivalents of Fmoc-Thr/Ser(αAc_3_GalNAc)-OH were sufficient to achieve quantitative coupling yields within 10 minutes of reaction time. There was no need in time-consuming double coupling procedures or potentially harmful microwave heating. Without microwave heating users of “traditional” synthesizers can perform glyco-economic yet speedy syntheses of *O*-glycopeptides. In addition, the absence of microwave heating avoids side reactions, such as the acetyl transfer reaction we observed. Remarkably high purities of crude products of 90–95% show that the new method allows very smooth solid-phase synthesis of mucin peptides encompassing up to 5 MUC5AC tandem repeats and carrying 10 GalNAc units.

(4) Native chemical ligation between two highly glycosylated peptides proceeded rapidly and cleanly also at the glycoamino acid junction.

With the presented method, solid-phase synthesis and purification of a peptide 40-mer with α-*O*-linked GalNAc residues on 25% of all amino acids takes 4 days. Within 2 days, subsequent solution steps, including thioester preparation, ligation, and desulfurization, are accomplished. We believe that these advances will further mucin research by facilitating access to a greater variety of GalNAcylated peptides in a short time.

## Data availability

The datasets supporting this article have been uploaded as part of the ESI.†

## Author contributions

O. S. formulated the research goals and aims. A. G. and E. K. conducted the research. C. S. performed measurements. A. G., E. K. and C. S. contributed to visualization of data. O. S., A. G., E. K. contributed to the original draft. All authors contributed to reviewing and editing.

## Conflicts of interest

There are no conflicts to declare.

## Supplementary Material

SC-015-D3SC05006H-s001
