# Supplementary material for: Rapid building block-economic synthesis of long, multi-O-GalNAcylated MUC5AC tandem repeat peptides
Source: Chem Sci. 2023 Dec 18;15(4):1297–305. doi: 10.1039/d3sc05006h (PMC10806717; doi:10.1039/d3sc05006h)
Supplement: SC-015-D3SC05006H-s001 [file SC-015-D3SC05006H-s001.pdf]

Electronic Supplementary Material (ESI) for Chemical Science.
This journal is © The Royal Society of Chemistry 2023

Table of Contents

1. General Information 3

2. Synthesis of Fmoc-Thr(*α*Ac_3GalNAc)-OH (**7t**) and Fmoc-Ser(*α*Ac_3GalNAc)-OH (**7s**)....4

2.1. Synthesis of galactosyl donors **2** and **5** 4

2.2. Synthesis of glycoamino acids 5

2.3 NMR-spectra 8

2.3.1 Phenyl 2-azido-2-deoxy-3,4,6-tri-O-acetyl-1-seleno-α-D-galactopyranoside (**2**)....8

2.3.2 (2-azido-2-deoxy-3,4,6-tri-O-acetyl-α-D-galactopyranosyl)-2,2,2-trichloroacetimidate (5). 8

2.3.3 Crude of glycosylation of Fmoc-Thr-O*t*Bu **3t** with phenylselenogalactosyl donor **2** (method **A**). 9

2.3.4 Crude of glycosylation of Fmoc-Thr-O*t*Bu **3t** with galactosyl trichloroacetimidate donor **5** (method **B**). 9

2.3.5 N-(Fluoren-9-ylmethoxycarbonyl)-O-(2-acetamido-2-deoxy-3,4,6-tri-Oacetyl-α-D-galactopyranosyl)-L-threonine tert-butyl ester (**6t**). 10

2.3.6 Crude of glycosylation of Fmoc-Ser-O*t*Bu **3s** with phenylselenogalactosyl donor **2** (method **A**). 10

2.3.7 Crude of glycosylation of Fmoc-Ser-O*t*Bu **3s** with galactosyl trichloroacetimidate donor **5** (method **B**). 11

2.3.8 N-(Fluoren-9-ylmethoxycarbonyl)-O-(2-acetamido-2-deoxy-3,4,6-tri-Oacetyl-α-D-galactopyranosyl)-L-serine tert-butyl ester (**6s**). 11

2.3.9 N-(Fluoren-9-ylmethoxycarbonyl)-O-(2-acetamido-2-deoxy-3,4,6-tri-Oacetyl-α-D-galactopyranosyl)-L-threonine (**7t**). 12

2.3.10 N-(Fluoren-9-ylmethoxycarbonyl)-O-(2-acetamido-2-deoxy-3,4,6-tri-Oacetyl-α-D-galactopyranosyl)-L-serine (**7s**). 12

3. Preparation of Tentagel R H_2N-NH-TRT resin 13

3.1 Loading of Tentagel R 2-Chlorotrityl resin with Fmoc-NH-NH_2 13

3.2 Quantifying Fmoc loading 13

4. Loading of the first amino acid to the H_2N-NH-TRT resin 14

4.1 Loading of Fmoc-Ser(O*t*Bu)-OH 14

4.2 Loading of Fmoc-Ser(αAc_3GalNAc)-OH (**7t**) in 2-MeTHF 14

5. Optimizing coupling of glycoamino acid 15

5.2 Optimizing coupling of Fmoc-Thr(αAc_3GalNAc)-OH (**7t**) in DMF solution (Table 4) 15

5.3 Optimizing coupling of Fmoc-Thr(αAc_3GalNAc)-OH (**7t**) in 2-MeTHF solution (Table 5) 17

6. Synthesis of glycopeptides 18

6.1 Glycopeptide hydrazides **11** and **12** 18

6.1.1 Ac-(AP**gT**TS**gT**TS)$_5$-NH-NH$_2$ (**11**) 18
6.1.2 Ac-(APTT**gS**TT**gS**)$_5$-NH-NH$_2$ (**12**) 18
6.2 Cysteine glycopeptide **13** 18
6.2.1 CP**gT**TS**gT**TS-(AP**gT**TS**gT**TS)$_4$ (**13**) 19
6.3 Experiments with glycopeptides containing O-acetyl-protected sugars 19
6.3.2 Synthesis of glycopeptide with O-acetyl protected sugars 20
6.3.3 Cleavage of O-acetyl protecting groups from sugars in solution 20
7. Native chemical ligation of glycopeptides 21
8. One-pot add-and-done desulfurization of glycopeptides 23
8.1 Ac-(AP**gT**TS**gT**TS)$_{10}$-NH$_2$ (**14**) 23
8.2 Ac-(APTT**gS**TT**gS**)$_5$-(AP**gT**TS**gT**TS)$_5$-NH$_2$ (**15**) 23
9. References 24

1. General Information

Commercially available compounds were used without further purification. Solvents for peptide synthesis were dried by using MBraun Solvent Purification System SPS 800. Dry solvents for glycoamino acid synthesis were obtained from Thermo Scientific (Schwerte, Germany). Molecular sieves 4Å were activated prior to reaction overnight at 250°C *in vacuo*.

Thin layer chromatography (TLC) was carried out on aluminium sheets coated with silica gel 60 F254 (Merck) (Darmstadt, Germany). TLC plates were inspected under UV light (λ = 254 nm) and developed by treatment with a mixture of 15% H_3PO_4 and orcinol (1.8 g/L) in EtOH–H_2O (5:95, v/v) followed by heating. Purification of compounds by flash chromatography was done on silica gel (0.04-0.63 mm, 60 Å) from Macherey-Nagel (Düren, Germany) using technical grade solvents.

NMR-spectra were recorded on a Bruker Avance II 300 MHz Spectrometer and referenced to the residual protonated solvent signal or/and tetramethylsilane.

Fmoc-SPPS was performed by using a Biotage Initiator+ Alstra synthesizer (Uppsala, Sweden).

Preparative HPLC purifications were carried out by using an Agilent 1100 Series system equipped with a Nucleodur C18 Gravity column (250 mm x 21 mm, 5 µm) from Macherey-Nagel with a binary mixture of A (0.1 % TFA, 1 % ACN, 98.9 % H_2O) and B (0.1 % TFA, 1 % H_2O, 98.9 % ACN) as a mobile phase (flow = 15 mL/min) in a linear gradient as described.

UPLC-MS measurements were performed by using an Acquity system from Waters and a BEH130 C18 column (2.1 x 50 mm, 1.7 µm; heater set on 50 °C) with a binary mixture of A (0.1 % TFA, 1 % ACN, 98.9 % H_2O) and B (0.1 % TFA, 1 % H_2O, 98.9 % ACN) as a mobile phase (flow = 0.5 mL/min) in a linear gradient as described.

High resolution ESI-MS spectra were recorded on a Waters H-class instrument equipped with a quaternary solvent manager, a Waters sample manager-FTN, a Waters PDA detector and a Waters column manager with an Acquity UPLC protein BEH C18 column (1.7 µm, 2.1 mm x 50 mm). Samples were eluted with a flow rate of 0.3 mL/min at 40 °C. The following gradient was used: A: 0.01% FA in H2O; B: 0.01% FA in MeCN. 5% B: 0-0.5 min; 5 to 95% B: 0.5-3.5 min; 95% B: 3.5-4 min. Mass analysis was conducted with a Waters XEVO G2-XS QTof analyzer and the recorded data was subsequently analyzed using the provided built-in software.

2. Synthesis of Fmoc-Thr(αAc$_3$GalNAc)-OH (**7t**) and Fmoc-Ser(αAc$_3$GalNAc)-OH (**7s**)

2.1. Synthesis of galactosyl donors **2** and **5**

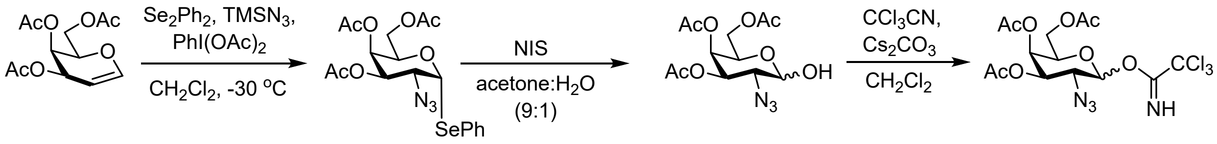

1 **2** **S1** **5**

Scheme S1. Synthesis of galactosyl donors **2** and **5**

Phenyl 2-azido-2-deoxy-3,4,6-tri-O-acetyl-1-seleno-α-D-galactopyranoside (**2**).

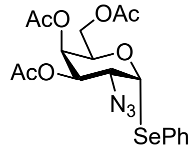

The stirred solution of tri-*O*-acetyl-galactal **1** (25 g, 91.91 mmol) and Se_2Ph_2 (17.21 g, 55.15 mmol) in anhydrous CH_2Cl_2 (384 mL) was cooled to –30°C, then $TMSN_3$ (24.2 mL, 183.82 mmol) and $PhI(OAc)_2$ (32.56 g, 101.1 mmol) were added sequentially. After $PhI(OAc)_2$ was dissolved, the temperature was maintained at –20°C for 16 hours. The mixture was diluted with CH_2Cl_2 and washed with sat. aq. $NaHCO_3$ (2x400 mL). The organic phase was dried by filtration through Na_2SO_4 and concentrated *in vacuo*. Crystallization from *i*PrOH, filtration and washing with petroleum ether gave 23.77 g (55%) of selenogalactoside **2** as a white solid. ^{1}H NMR data are in accord with the literature.[1]

(2-azido-2-deoxy-3,4,6-tri-O-acetyl-α-D-galactopyranosyl)-2,2,2-trichloroacetimidate (**5**).

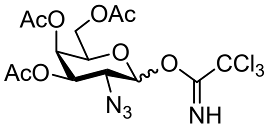

To a stirred solution of selenogalactoside **2** (19.27 g, 40.97 mmol) in a mixture of acetone: water (9:1, v/v, 410 mL) was added NIS (27.7 g, 122.9 mmol). After one hour stirring, the mixture was diluted with CH_2Cl_2 (80 mL) and washed with 1 M aq. $Na_2S_2O_3$ (2x400 mL) and sat. aq. $NaHCO_3$ (1x400 mL). The organic phase was dried by filtration through Na_2SO_4 and concentrated *in vacuo*. The residue was purified by column chromatography [cyclohexene: EtOAc (2:1)], giving hemiacetal **S1** (13.03 g, quant.).
To the solution of obtained **S1** (13.03 g, 40.55 mmol) in anhydrous CH_2Cl_2 (400 mL), trichloroacetonitrile (41 mL, 4.11 mol) and Cs_2CO_3 (4.01 g) were added under stirring. After one hour, the mixture was filtrated and washed with sat. aq. $NaHCO_3$ (2x400 mL). The organic phase was dried by filtration through Na_2SO_4 and concentrated *in vacuo*. The residue was purified by column chromatography [cyclohexene:EtOAc (2:1) + 0.1% Et_3N], giving 16.37 g (84%) of galactosyl trichloroacetimidate **5** as α:β (13:1) mixture; a syrup. ^{1}H NMR data are in accord with the literature.[2]

2.2. Synthesis of glycoamino acids

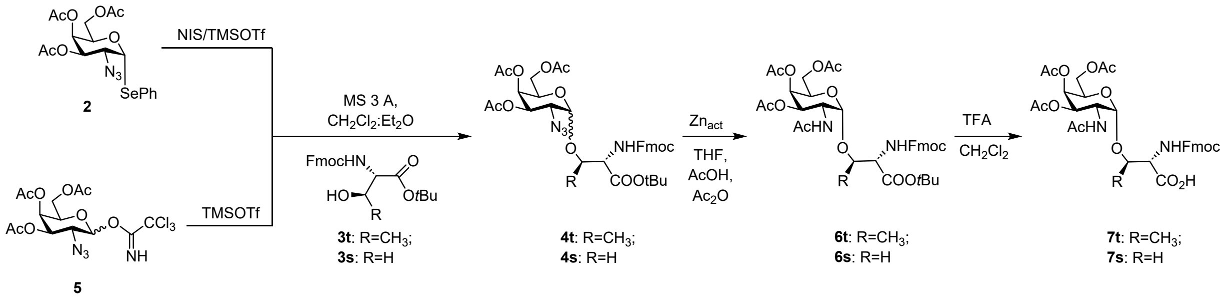

Scheme S2. Synthesis of glycoamino acids Fmoc-Thr(*α*Ac_3GalNAc)-OH (**7t**) and Fmoc-Ser(*α*Ac_3GalNAc)-OH (**7s**)

N-(Fluoren-9-ylmethoxycarbonyl)-O-(2-acetamido-2-deoxy-3,4,6-tri-Oacetyl-α-D-galactopyranosyl)-L-threonine tert-butyl ester (**6t**).

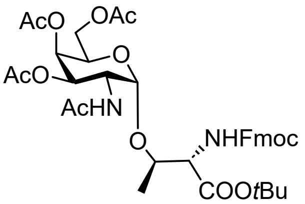

A: A carefully dried[a] mixture of galactosyl donor **2** (1.03 g, 2.19 mmol) and Fmoc-Thr-O*t*Bu **3t** (1.05 g, 2.63 mmol, 1.2 eq.) was dissolved in dry CH_2Cl_2–Et_2O mixture (1:1, v/v, 18 mL) and molecular sieves 3 Å (1.8 g) were added. After 30 min, the stirred mixture was cooled to –10°C and NIS (591.5 mg, 2.63 mmol) and TMSOTf (40 µL, 0.22 mmol) were added. The mixture was stirred for 10 min and quenched with one drop of Et_3N. The mixture was filtered through a Celite pad with CH_2Cl_2, and the filtrate was washed with 1 M aq. $Na_2S_2O_3$ (1x200 mL) and sat. aq. $NaHCO_3$ (1x200 mL). The organic phase was dried by filtration through Na_2SO_4 and concentrated *in vacuo.*[b] The residue was purified by column chromatography [cyclohexene:CH_2Cl_2: EtOAc (1:1:0→4.5:4.5:1)], giving α/β-mixture **4t** with unidentifiable impurities (809.8 mg).
To a stirred solution of the obtained compound **4t** (809.8 mg) in a mixture of THF, AcOH and Ac_2O (3:2:1, v/v, 9 mL) activated zinc dust[3] (727.5 mg, 11.12 mmol) was added. Stirring was continued for an hour, then the zinc dust was filtered off and washed with CH_2Cl_2. The combined organic solutions were washed with sat. aq. $NaHCO_3$ (2x200 mL), the organic phase was dried by filtration through Na_2SO_4 and concentrated *in vacuo*. The residue was purified by column chromatography [cyclohexene: EtOAc (2:3)], giving 382 mg (24%) of glycothreonine tert-butyl ester **6t**; white solid. ^{1}H NMR data are in accord with the literature.[4]

B: A carefully dried[a] mixture of galactosyl donor **5** (9.31 g, 19.8 mmol) and Fmoc-Thr-O*t*Bu **3t** (8.66 g, 21.78 mmol, 1.1 eq.) was dissolved in dry CH_2Cl_2–Et_2O mixture (1:1, v/v, 198 mL) and molecular sieves 3 Å (19.8 g) were added. After 30 min, reaction was cooled to 0°C and TMSOTf (0.465 mL, 2.57 mmol) was added. The mixture was stirred for an hour at 0°C and quenched with one drop of Et_3N. The mixture was filtered through a Celite pad with CH_2Cl_2, and the filtrate was washed with 1M aq. HCl (1x300 mL) sat. aq. $NaHCO_3$ (1x300 mL). The organic phase was dried by filtration through Na_2SO_4 and concentrated *in vacuo*.[c] The residue was purified by column

[a]Toluene co-evaporation 3 times.
[b]NMR-sample was taken to assess α/β-ratio of crude (page S9).
[c] NMR-sample was taken to assess α/β-ratio of crude (page S9).

chromatography [cyclohexene:CH_2Cl_2: EtOAc (1:1:0→4.5:4.5:1)], giving α/β-mixture of **4t** with unidentifiable impurities (13.3 g).
To the solution of the obtained compound **4t** (13.3 g) in a mixture of THF, AcOH and Ac_2O (3:2:1, v/v, 146 mL) activated zinc dust[3] (11.87g, 181.5 mmol) was added. Stirring was continued for an hour, then the zinc dust was filtered off and washed with CH_2Cl_2. The combined organic solutions were washed with sat. aq. $NaHCO_3$ (4x300 mL), the organic phase was dried by filtration through Na_2SO_4 and concentrated *in vacuo*. The residue was purified by column chromatography [cyclohexene: EtOAc (2:3)], giving 6.77 g (47%) of glycothreonine tert-butyl ester **6t**; white solid. All spectral characteristics were completely identical to the compound **6t** obtained by method **A**.

N-(Fluoren-9-ylmethoxycarbonyl)-O-(2-acetamido-2-deoxy-3,4,6-tri-Oacetyl-α-D-galactopyranosyl)-L-serine tert-butyl ester (**6s**).

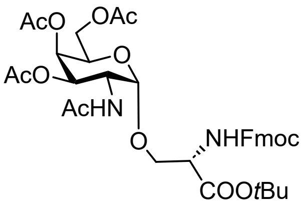

A: A carefully dried[a] mixture of galactosyl donor **2** (343 mg, 0.73 mmol, 1.2 eq.) and Fmoc-Ser-O*t*Bu **3s** (233 mg, 0.61 mmol) was dissolved in dry CH_2Cl_2–Et_2O mixture (1:1, v/v, 7.2 mL) and molecular sieves 3 Å (720 mg) were added. After 30 min, the stirred mixture was cooled to –10°C and NIS (196.9 mg, 0.88 mmol) and TMSOTf (13 µL, 73 µmol) were added. The mixture was stirred for 10 min and quenched with one drop of Et_3N. The mixture was filtered through a Celite pad with CH_2Cl_2, and the filtrate was washed with 1 M aq. $Na_2S_2O_3$ (1x150 mL) and sat. aq. $NaHCO_3$ (1x100 mL). The organic phase was dried by filtration through Na_2SO_4 and concentrated *in vacuo.*[d] The residue was purified[e] by column chromatography [cyclohexene:CH_2Cl_2:EtOAc (1:1:0→4.5:4.5:1)], giving α-anomer of **4s** (200 mg, with unidentifiable impurities).
To a stirred solution of the obtained compound **4s** in a mixture of THF, AcOH and Ac_2O (3:2:1, v/v, 4.5 mL) activated zinc dust[3] (354.2 mg, 5.42 mmol) was added. Stirring was continued for an hour, then the zinc dust was filtered off and washed with CH_2Cl_2. The combined organic solutions were washed with sat. aq. $NaHCO_3$ (2x100 mL), the organic phase was dried by filtration through Na_2SO_4 and concentrated *in vacuo*. The residue was purified by column chromatography [cyclohexene: EtOAc (2:3)], giving 113.1 mg (26%) **6s**; white solid. ^{1}H NMR data are in accord with the literature.[4]

B: A carefully dried[a] mixture of galactosyl donor **5** (3.18 g, 6.77 mmol, 1.2 eq.) and Fmoc-Ser-O*t*Bu **3s** (2.16 g, 5.64 mmol) was dissolved in dry CH_2Cl_2–Et_2O mixture (1:1, v/v, 68 mL) and molecular sieves 3 Å (680 mg) were added. After 30 min, the mixture was cooled to 0°C and TMSOTf (159 µL, 0.88 mmol) was added. The mixture was stirred for an hour at 0°C and quenched with one drop of Et_3N. The mixture was filtered through a Celite pad with CH_2Cl_2, and the filtrate was washed with 1M aq. HCl (1x250 mL) and sat. aq. $NaHCO_3$ (1x250 mL). The organic phase was dried by filtration through Na_2SO_4 and concentrated *in vacuo*.[f] The residue was purified[g] by column chromatography [cyclohexene: CH_2Cl_2: EtOAc (1:1:0→4.5:4.5:1)], giving α-anomer (2.37 g, with unidentifiable impurities).

[d]NMR-sample was taken to assess α/β-ratio of crude (page S10).
[e]In case of Fmoc-Ser(Ac_3GalN_3)-OtBu, it is crucial to separate α- from β-anomer as they cannot be separated in the next step. The column was performed twice in this case.
[f]NMR-sample was taken to assess α/β-ratio of crude (page S11).
[g]The column was performed twice in this case.

To the solution of the obtained compound **4s** (2.37 g) in a mixture of THF, AcOH and Ac_2O (3:2:1, v/v, 30 mL) activated zinc dust[3] (2.45 g, 37.44 mmol) was added. Stirring was continued for an hour, then the zinc dust was filtered off and washed with CH_2Cl_2. The combined organic solutions were washed with sat. aq. $NaHCO_3$ (3x250 mL), the organic phase was dried by filtration through Na_2SO_4 and concentrated *in vacuo*. The residue was purified by column chromatography [cyclohexene: EtOAc (2:3)], giving 2.05 g (51%) of **6s**; white solid. All spectral characteristics were completely identical to the compound **6s** obtained by method **A**.

N-(Fluoren-9-ylmethoxycarbonyl)-O-(2-acetamido-2-deoxy-3,4,6-tri-Oacetyl-α-D-galactopyranosyl)-L-threonine (**7t**).

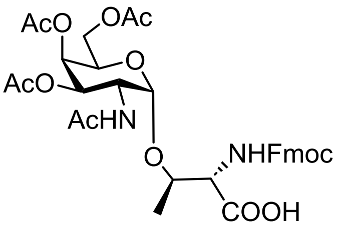

Fmoc-protected glycothreonine tert-butyl ester **6t** (4.016 g, 5.53 mmol) was dissolved in mixture of CH_2Cl_2 and TFA (1:1, v/v, 87.5 mL) and stirred for 4 hours. The reaction mixture was concentrated and co-evaporated 3 times with toluene. The crude was purified by column chromatography [CH_2Cl_2: MeOH (2%)], giving 2.78 g (75 %) of glycothreonine **7t** as light beige solid. ^{1}H NMR data are in accord with the literature.[4]

N-(Fluoren-9-ylmethoxycarbonyl)-O-(2-acetamido-2-deoxy-3,4,6-tri-Oacetyl-α-D-galactopyranosyl)-L-serine (**7s**).

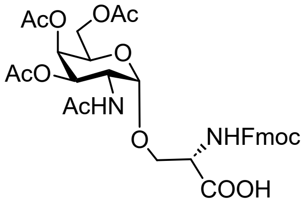

Fmoc-protected glycoserine tert-butyl ester **6s** (1.45 g, 2.038 mmol) was dissolved in mixture of CH_2Cl_2 and TFA (1:1, v/v, 23 mL) and stirred for 4 hours. The reaction mixture was concentrated and co-evaporated 3 times with toluene. The crude was purified by column chromatography [CH_2Cl_2: MeOH (2%)], giving 936.9 mg (70%) of glycoserine **7s** as a light beige solid. ^{1}H NMR data are in accord with the literature.[4]

2.3 NMR-spectra

2.3.1 Phenyl 2-azido-2-deoxy-3,4,6-tri-O-acetyl-1-seleno-α-D-galactopyranoside (**2**).

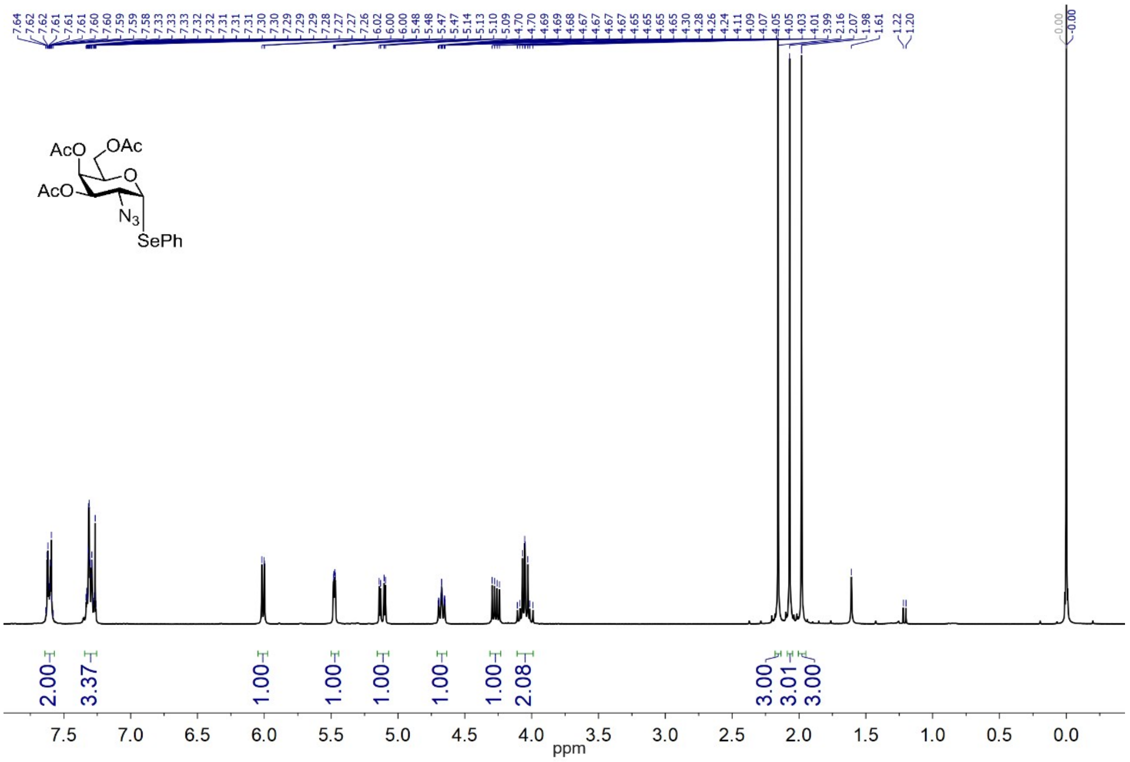

300 MHz, $CDCl_3$

2.3.2 (2-azido-2-deoxy-3,4,6-tri-O-acetyl-α-D-galactopyranosyl)-2,2,2-trichloroacetimidate (5).

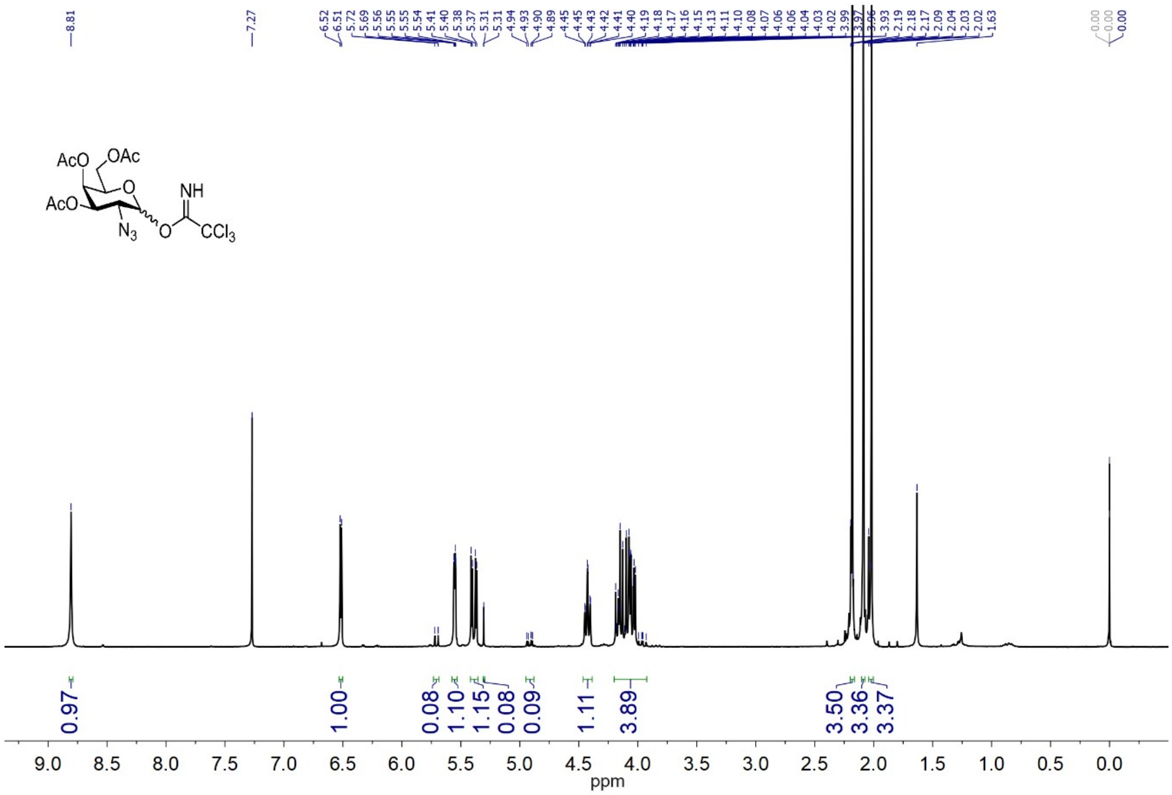

300 MHz, $CDCl_3$

2.3.3 Crude of glycosylation of Fmoc-Thr-O*t*Bu **3t** with phenylselenogalactosyl donor **2** (method **A**).

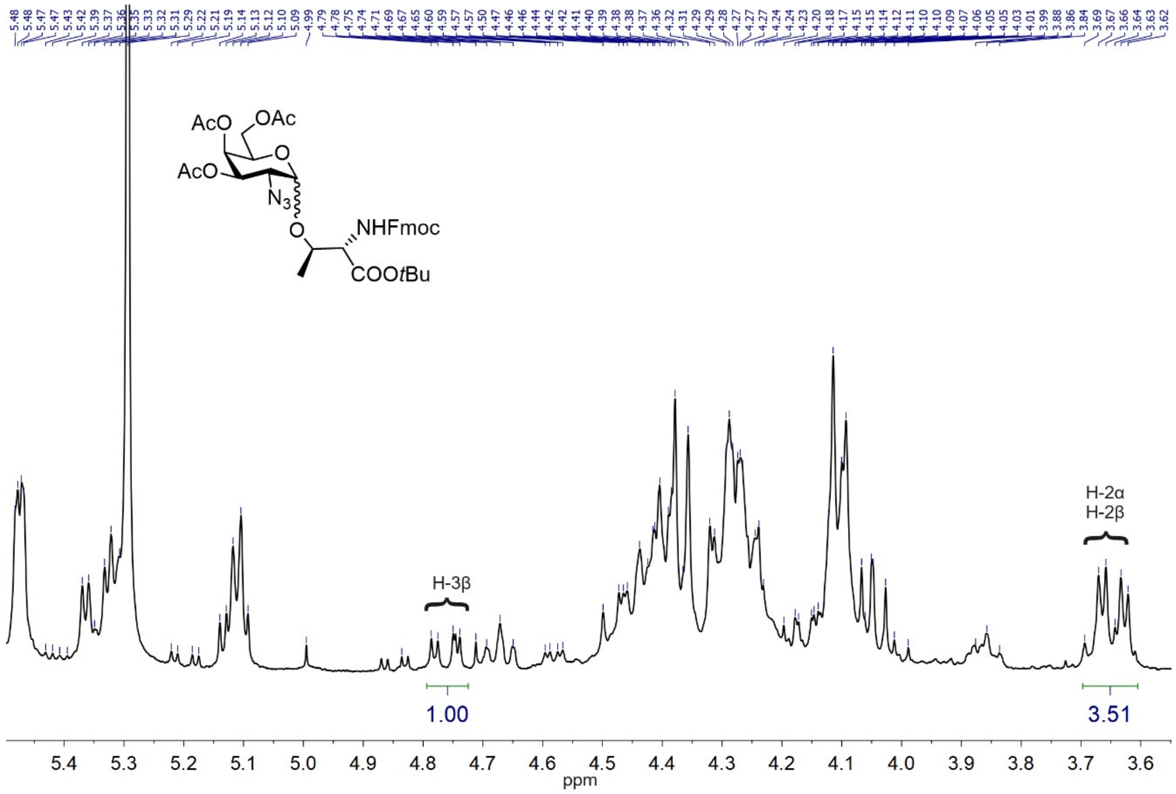

300 MHz, $CDCl_3$

2.3.4 Crude of glycosylation of Fmoc-Thr-O*t*Bu **3t** with galactosyl trichloroacetimidate donor **5** (method **B**).

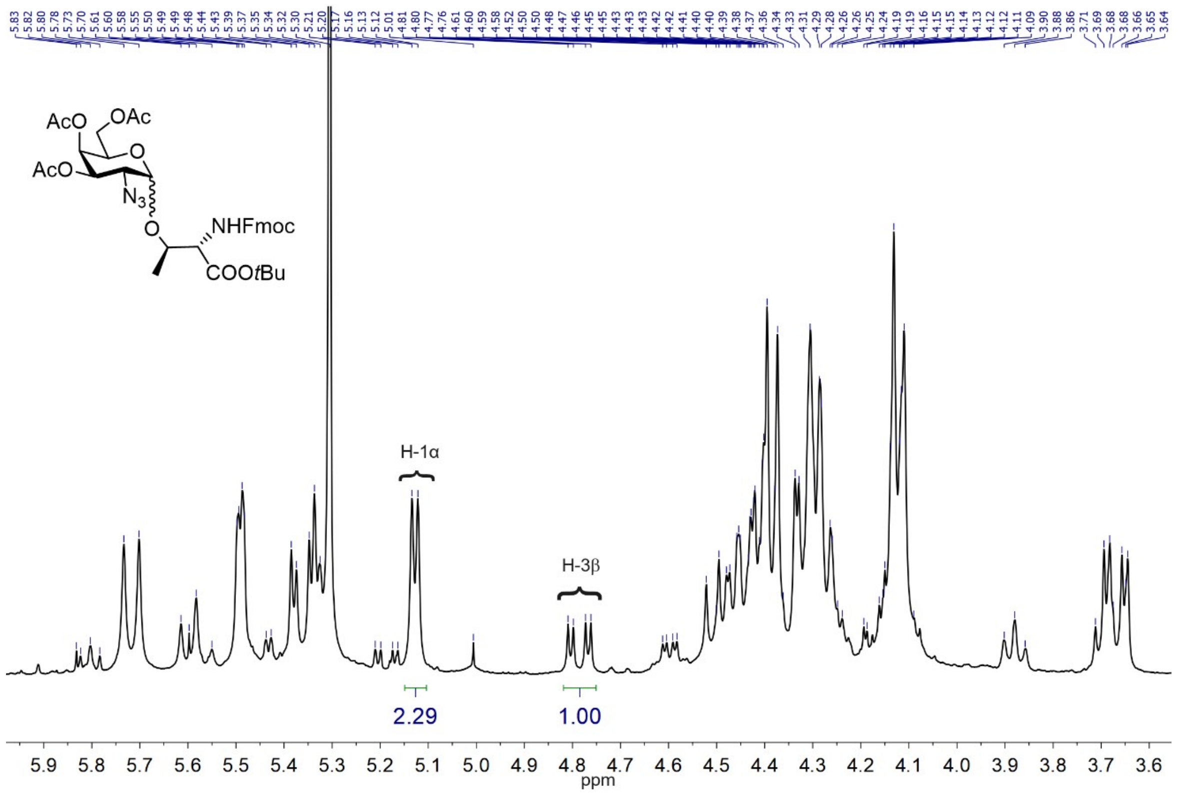

300 MHz, $CDCl_3$

2.3.5 N-(Fluoren-9-ylmethoxycarbonyl)-O-(2-acetamido-2-deoxy-3,4,6-tri-Oacetyl-α-D-galactopyranosyl)-L-threonine tert-butyl ester (**6t**).

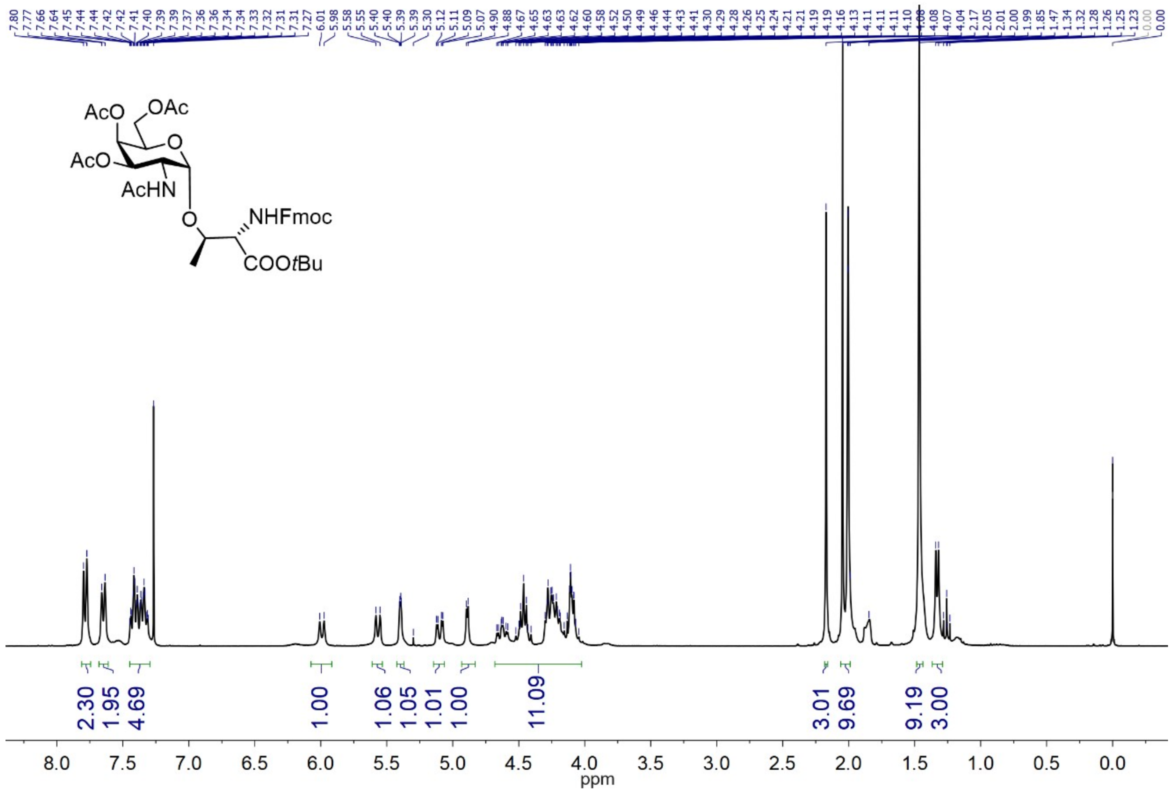

300 MHz, $CDCl_3$

2.3.6 Crude of glycosylation of Fmoc-Ser-O*t*Bu **3s** with phenylselenogalactosyl donor **2** (method **A**).

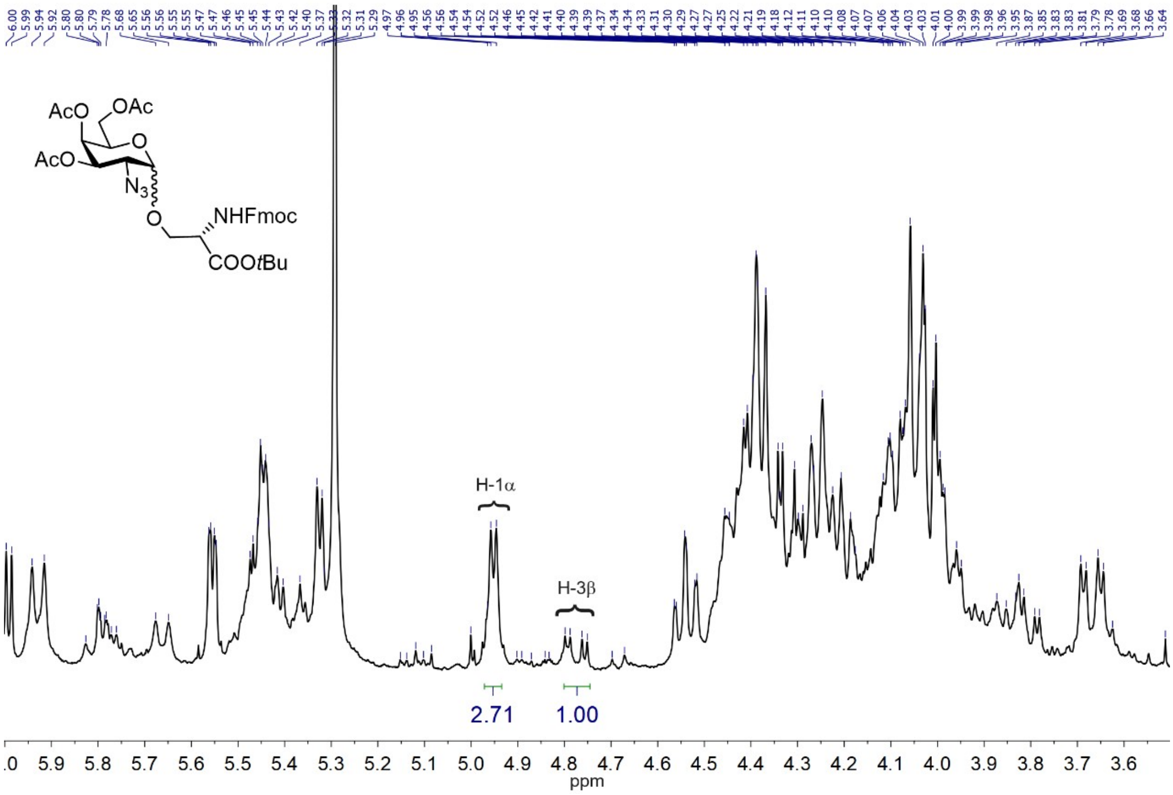

300 MHz, $CDCl_3$

2.3.7 Crude of glycosylation of Fmoc-Ser-O*t*Bu **3s** with galactosyl trichloroacetimidate donor **5** (method **B**).

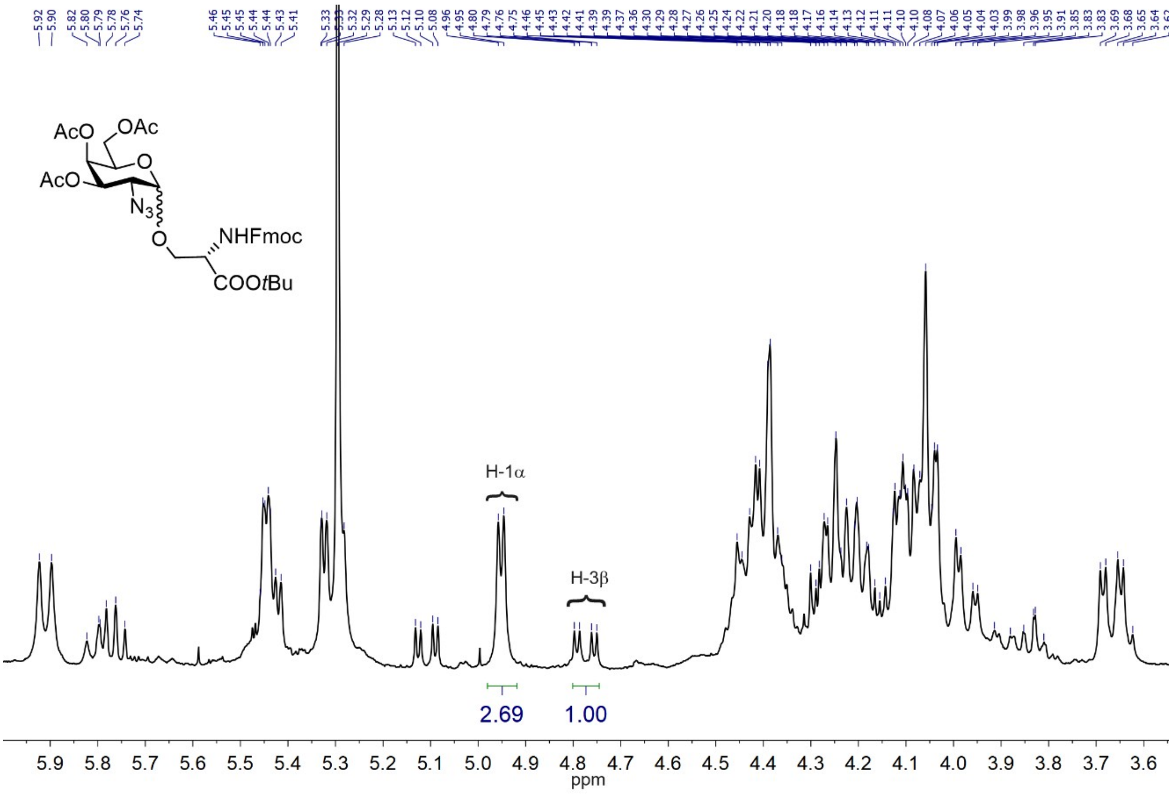

300 MHz, $CDCl_3$

2.3.8 N-(Fluoren-9-ylmethoxycarbonyl)-O-(2-acetamido-2-deoxy-3,4,6-tri-Oacetyl-α-D-galactopyranosyl)-L-serine tert-butyl ester (**6s**).

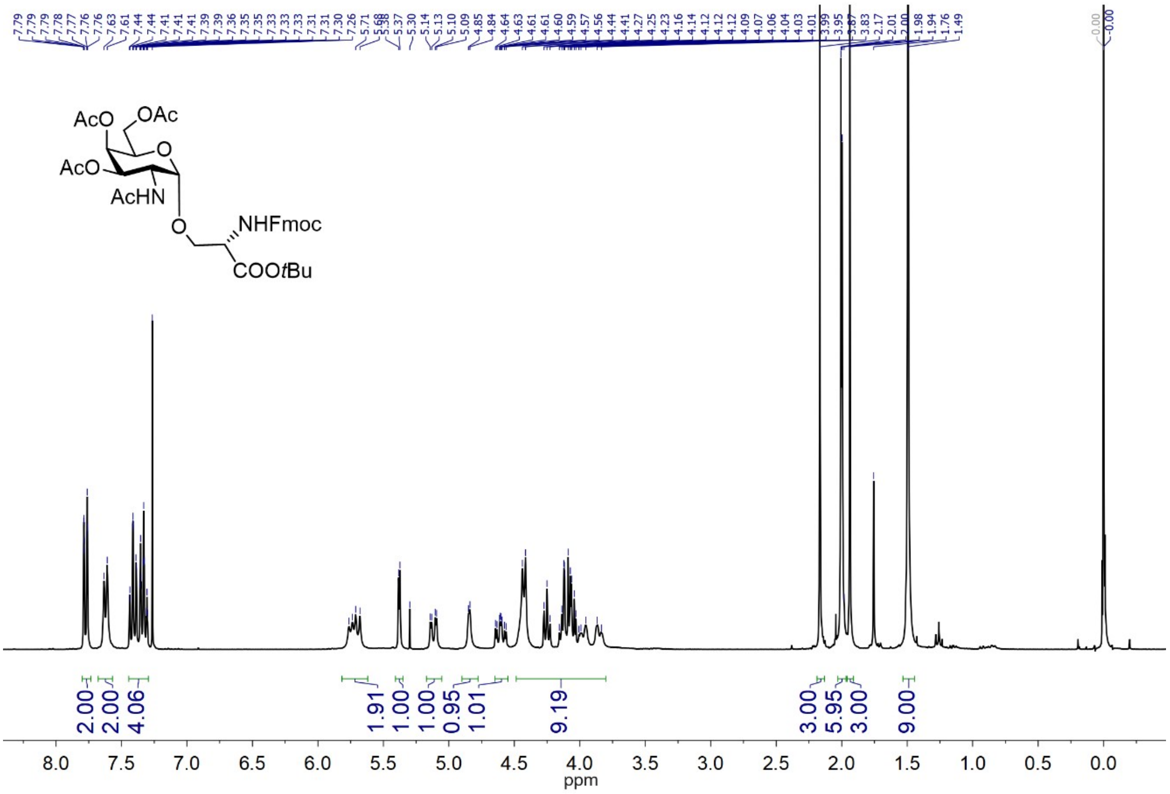

300 MHz, $CDCl_3$

2.3.9 N-(Fluoren-9-ylmethoxycarbonyl)-O-(2-acetamido-2-deoxy-3,4,6-tri-Oacetyl-α-D-galactopyranosyl)-L-threonine (**7t**).

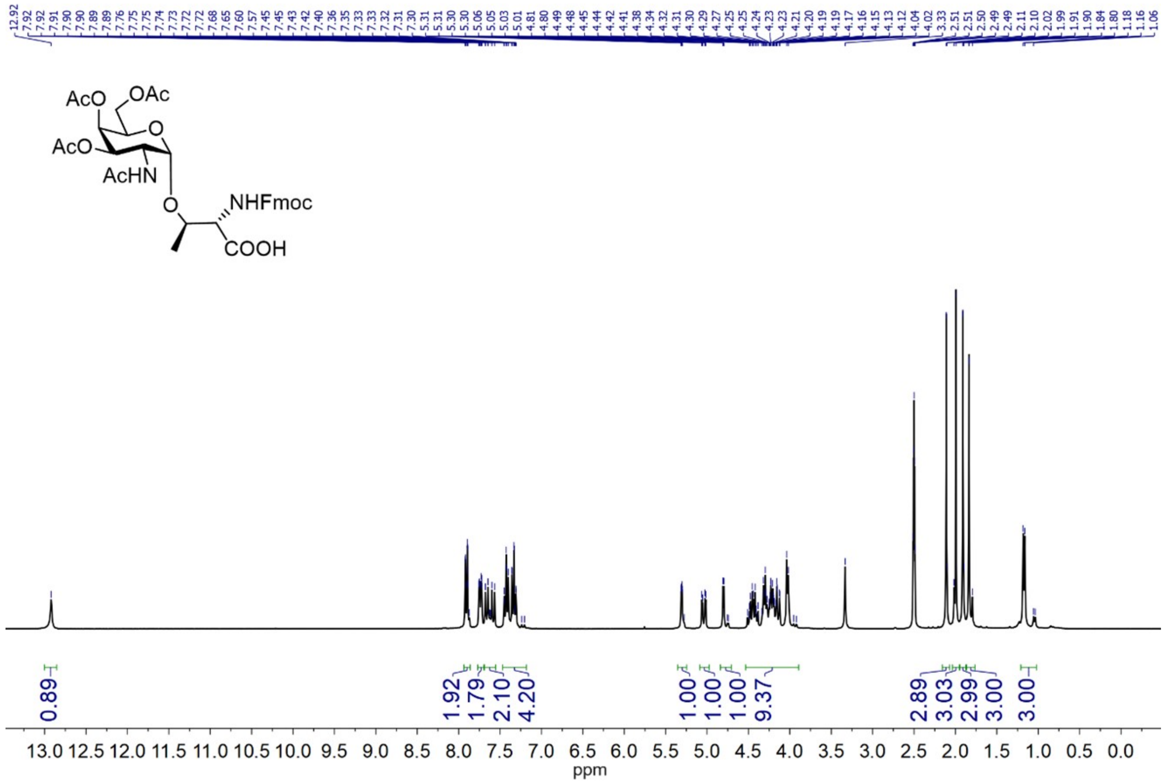

300 MHz, DMSO-d6

2.3.10 N-(Fluoren-9-ylmethoxycarbonyl)-O-(2-acetamido-2-deoxy-3,4,6-tri-Oacetyl-α-D-galactopyranosyl)-L-serine (**7s**).

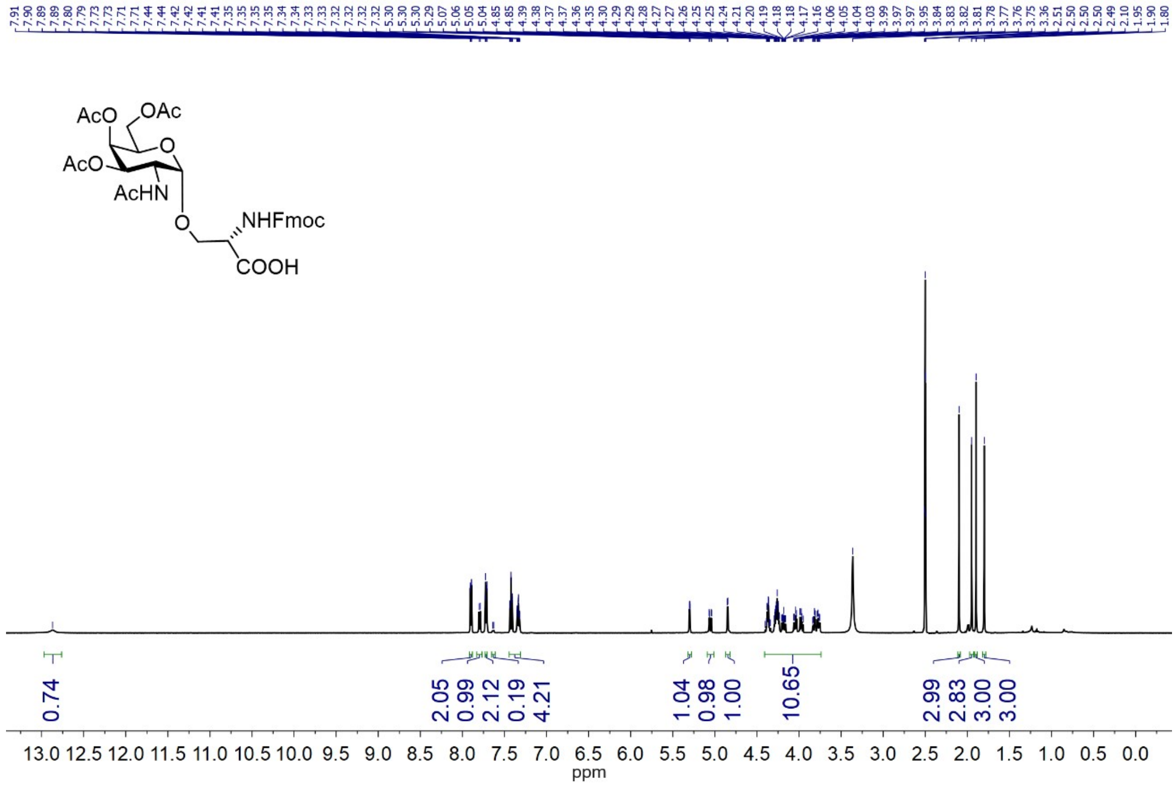

300 MHz, DMSO-d6

3. Preparation of Tentagel R H_2N-NH-TRT resin

3.1 Loading of Tentagel R 2-Chlorotrityl resin with Fmoc-NH-NH_2

The resin (~0.17 mmol/g) was transferred into a syringe equipped with a filter frit and allowed to swell in anhydrous CH_2Cl_2 (10 min). Hydrolysed trityl residues were reactivated by addition of $SOCl_2$ in anhydrous CH_2Cl_2 (10% v/v) for 2x30 min. The resin was washed (5x anhydrous CH_2Cl_2, 5xDIPEA in anhydrous CH_2Cl_2 (5% v/v), 5x anhydrous CH_2Cl_2).

Reactivated trityl resin was allowed to swell in CH_2Cl_2:DMF (1:1, v/v) for 30 min and subsequently treated with Fmoc-NH-NH_2[5] (10 eq.) in anhydrous DMSO (0.5M). After 1 h the resin was washed (5x CH_2Cl_2), capped with a solution of DIPEA (50 eq.) in MeOH: CH_2Cl_2 (1:3, v/v + 5% DiPEA) for 10 min and then washed again (5xCH_2Cl_2, 5xDMF). The Fmoc-group was removed by treatment of the resin with a solution of 20 % piperidine in DMF (2x 5 min), the initial loading estimated by the UV-absorbance of the piperidine-fulvene adduct (λ = 301 nm, ε = 7800 M^{-1} cm^{-1}), and the resin was washed (5xDMF, 5xCH_2Cl_2, 5xDMF). As estimated. the loading procedure gave 96% yield.

3.2 Quantifying Fmoc loading

The resin with N-terminal Fmoc group was swollen in 2 mL of piperidine:DMF solution (1:4, v/v) and placed on a shaker for 5 minutes. The liquid was drained in a 10 mL glass flask, equipped with a sept, and vacuum was applied to ensure all liquid is drained. Treatment was repeated once more.

In a glass cuvette, 990 µL of the same piperidine:DMF solution was added. From the solution containing piperidine-fulvene adduct, 10 µL was withdrawn and added to the glass cuvette. The mixture was shaken for 30 seconds.

UV-absorbance value was measured, and converted to µmol value according to the following formula:

$$n = \frac{A301 * V1 * V2}{E * V3}$$

where A301 = UV-absorbance value of the piperidine-fulvene adduct obtained after measurement;

V1 = total amount of piperidine:DMF solution used to treat resin (4000 µL)

V2 = volume of glass cuvette (1000 µL)

E = extinction coefficient of piperidine-fulvene adduct (7800 M^{-1} cm^{-1})

V3 = volume of piperidine:DMF solution taken for the measurement (10 µL)

4. Loading of the first amino acid to the H_2N-NH-TRT resin

4.1 Loading of Fmoc-Ser(O*t*Bu)-OH

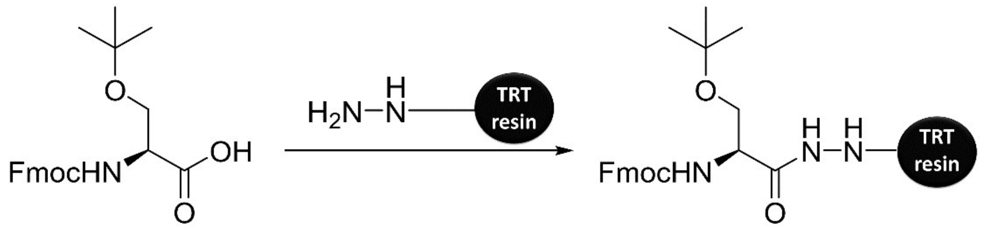

Scheme S3. Loading of Fmoc-Ser(O*t*Bu)-OH on hydrazine resin

Loading in DMF solution (Conditions 1 – 4 in Table 2)
10 eq. of Fmoc-Ser(O*t*Bu)-OH, 10 eq. of activating reagent and, if added, Oxyma, and 20 eq. of DiPEA were dissolved in DMF to achieve 0.5M concentration of all reagents. This mixture was added to H_2N-NH-TRT resin in a fritted syringe. At indicated time points, liquid was drained, and the resin was washed (5xDMF, 5xCH_2Cl_2, 5xDMF). To cap unreacted sites, resin was treated with DMF:Ac_2O:DiPEA (70:20:10, v/v/v) mixture for 5 min. The yields of reaction were estimated by the UV-absorbance of the piperidine-fulvene adduct (λ = 301 nm, ε = 7800 M^{-1} cm^{-1}).

Loading in 2-MeTHF solution (Conditions 5 – 6 in Table 2)
10 eq. of Fmoc-Ser(O*t*Bu)-OH, 10 eq. of Oxyma, 10 eq. of DIC were dissolved in 2-MeTHF to achieve 0.5M concentration of all reagents. After preactivation for 2 min, liquid was filtered from formed diisopropylurea crystals, and the filtrate added to H_2N-NH-TRT resin in a fritted syringe. At indicated time points, liquid was drained, and the resin was washed (5xDMF, 5xCH_2Cl_2, 5xDMF). To cap unreacted sites, resin was treated with DMF:Ac_2O:DiPEA (70:20:10, v/v/v) mixture for 5 min. The yields of reaction were estimated by the UV-absorbance of the piperidine-fulvene adduct (λ = 301 nm, ε = 7800 M^{-1} cm^{-1}).

4.2 Loading of Fmoc-Ser(αAc$_3$GalNAc)-OH (**7t**) in 2-MeTHF

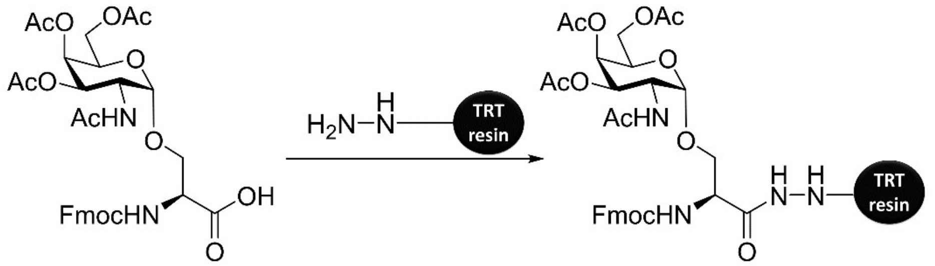

Scheme S4. Loading of Fmoc-Ser(αAc$_3$GalNAc)-OH on hydrazine resin

Indicated amount of Fmoc-Ser(αAc$_3$GalNAc)-OH, DIC and Oxyma were dissolved in 2-MeTHF. The final concentration of all compounds was 50 mM. After preactivation for 3 minutes, liquid was filtered from formed diisopropylurea crystals, and the filtrate was added to H_2N-NH-TRT resin in a fritted syringe. At indicated time points, liquid was drained, and the resin was washed (5xDMF, 5xCH_2Cl_2, 5xDMF). To cap unreacted sites, resin was treated with DMF:Ac_2O:DiPEA (70:20:10, v/v/v) mixture for 5 min. The yields of reaction were estimated by the UV-absorbance of the piperidine-fulvene adduct (λ = 301 nm, ε = 7800 M^{-1} cm^{-1}).

5. Optimizing coupling of glycoamino acid

5.1 Solid-phase synthesis of peptide **8***:

Automated synthesis was performed at 40 µmol scale on Rink Amide resin (0.20 mmol/g) by using a Biotage Initiator+ Alstra synthesizer (Uppsala, Sweden).

Coupling: Fmoc-protected amino acids (5 eq.) were transferred to the resin, followed by DIC and Oxyma (5 eq. each). The final concentration of amino acid was 167 mM. Reaction temperature was elevated to 90°C (coupling time = 1 min). The resin was washed with 2x 2 mL of DMF, each washing was performed for 1 minute.

Fmoc removal: The resin was treated with 20% piperidine in DMF for 1 min at 90°C. The resin was washed with 3x 2 mL of DMF, each washing was performed for 1 minute.

Capping: The resin was treated with DMF:Ac_2O:DiPEA (70:20:10, v/v/v) for 5 min at RT. The resin was washed with 2x 2 mL of DMF, each washing was performed for 1 minute.

TFA cleavage: Prior to TFA cleavage the Fmoc group was removed as described. The resin was washed with CH_2Cl_2 and dried under vacuum. Then 5 mL of a mixture of TFA:TIS:H_2O (96:2:2, v/v/v) was added to the resin. After 2 h the cleavage cocktail was collected by filtration, the resin was washed once with 5 mL of TFA and the combined filtrates were concentrated under air flow.

Peptide work-up: To the remaining residue cold Et_2O (9-fold volume) was added and the suspension was centrifuged (4000 rpm, 5 min). Afterwards the ether phase was decanted. The peptide precipitate was dissolved in Milli-Q water.

5.2 Optimizing coupling of Fmoc-Thr(αAc$_3$GalNAc)-OH (**7t**) in DMF solution (Table 4)

Condition 1: Automated coupling was performed with 10 µmol of **8*** by using the Biotage Initiator+ Alstra synthesizer. Fmoc-protected glycoamino acid **7t** was dissolved in Oxyma/DMF solution (55 mM) and combined with DIC until a final concentration of 50 mM **7t**/DIC/Oxyma was obtained. Reaction temperature was elevated to 75°C and coupling was performed for 5 min. The resin was washed (5xDMF, 5xCH_2Cl_2). *No capping was performed.* Fmoc removal and TFA cleavage were performed as described under 5.1.

Conditions 2-14: Couplings were performed manually with 4 mg (~0.4 µmol) of **8***. The indicated amount of Fmoc-Thr(αAc$_3$GalNAc)-OH, HATU, HOAt, and base were dissolved in DMF to achieve a final concentration of 50 mM glycoamino acid. The solution was added to peptide resin **8*** in a fritted syringe. At indicated time points, liquid was drained, and the resin was washed (5xDMF, 5xCH_2Cl_2, 5xDMF). *No capping was performed.* Fmoc removal was performed manually by treating the resin with 20 % piperidine in DMF for 10 min. The resin was washed (5xDMF, 5xCH_2Cl_2). TFA cleavage was performed as described under 5.1. The yields of reactions were estimated by integrating peaks in UPLC chromatograms.

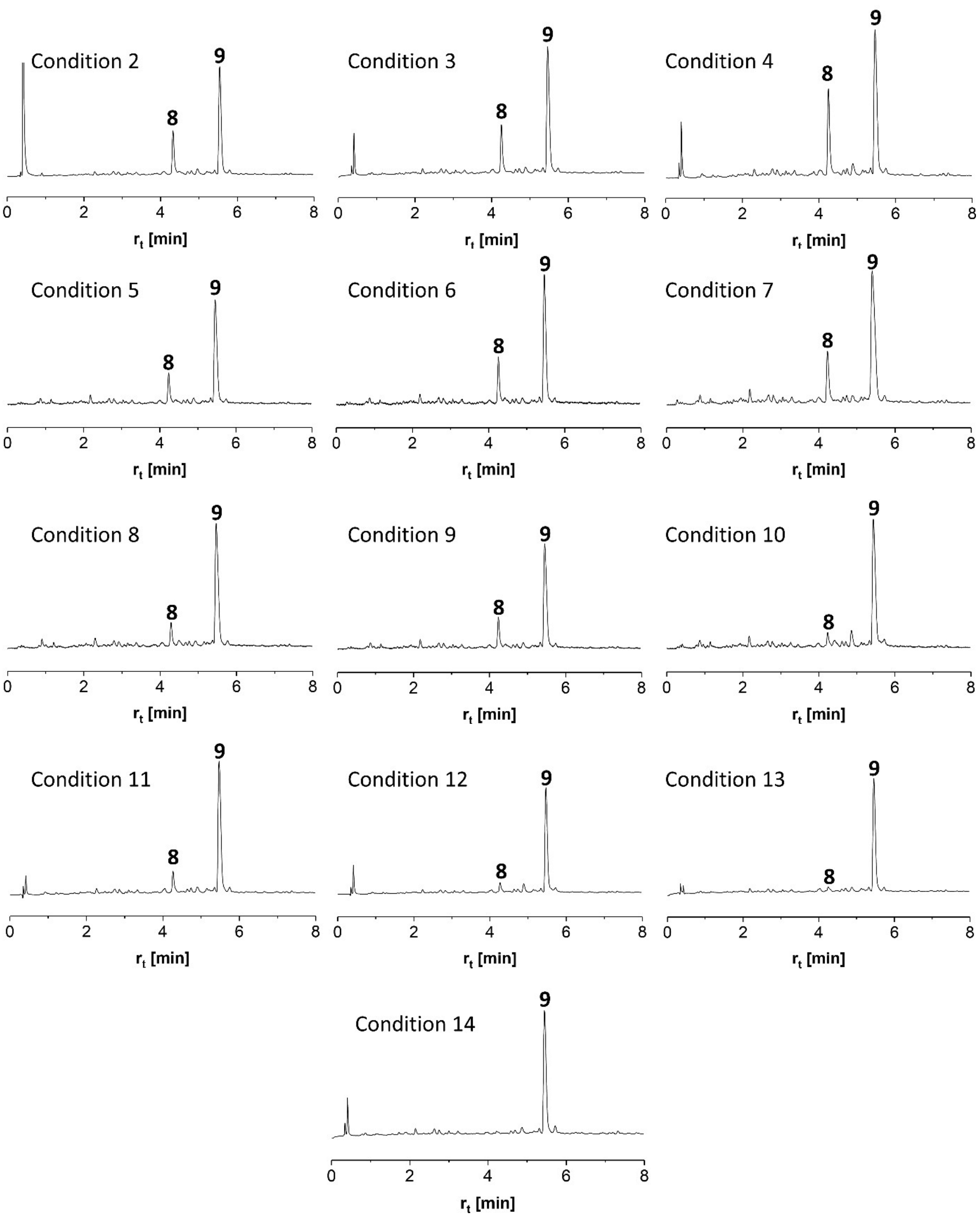

Figure S5. UPLC analysis of peptide **9**, obtained after coupling of **7t** to **8*** in DMF with conditions from Table 4. Gradient 15-30% ACN in Water + 0.1% TFA. λ = 210 nm

5.3 Optimizing coupling of Fmoc-Thr(αAc$_3$GalNAc)-OH (**7t**) in 2-MeTHF solution (Table 5)

Couplings were performed manually on 4 mg (~0.4 µmol) resin-bound peptide **8***. The indicated amount of Fmoc-Thr(αAc$_3$GalNAc)-OH, DIC and Oxyma were dissolved in 2-MeTHF to achieve 50 mM concentration. After 3 minutes, formed diisopropylurea crystals were removed by filtration. The filtrate was added to resin-bound peptide **8***. At indicated time points, liquid was drained, and the resin was washed (5xDMF, 5xCH_2Cl_2, 5xDMF). *No capping was performed*. Fmoc removal was performed manually by treating the resin with 20 % piperidine in DMF for 10 min. The resin was washed (5xDMF, 5xCH_2Cl_2). TFA cleavage was performed as described under 5.1. The yields of reactions were estimated by integrating peaks in UPLC chromatograms.

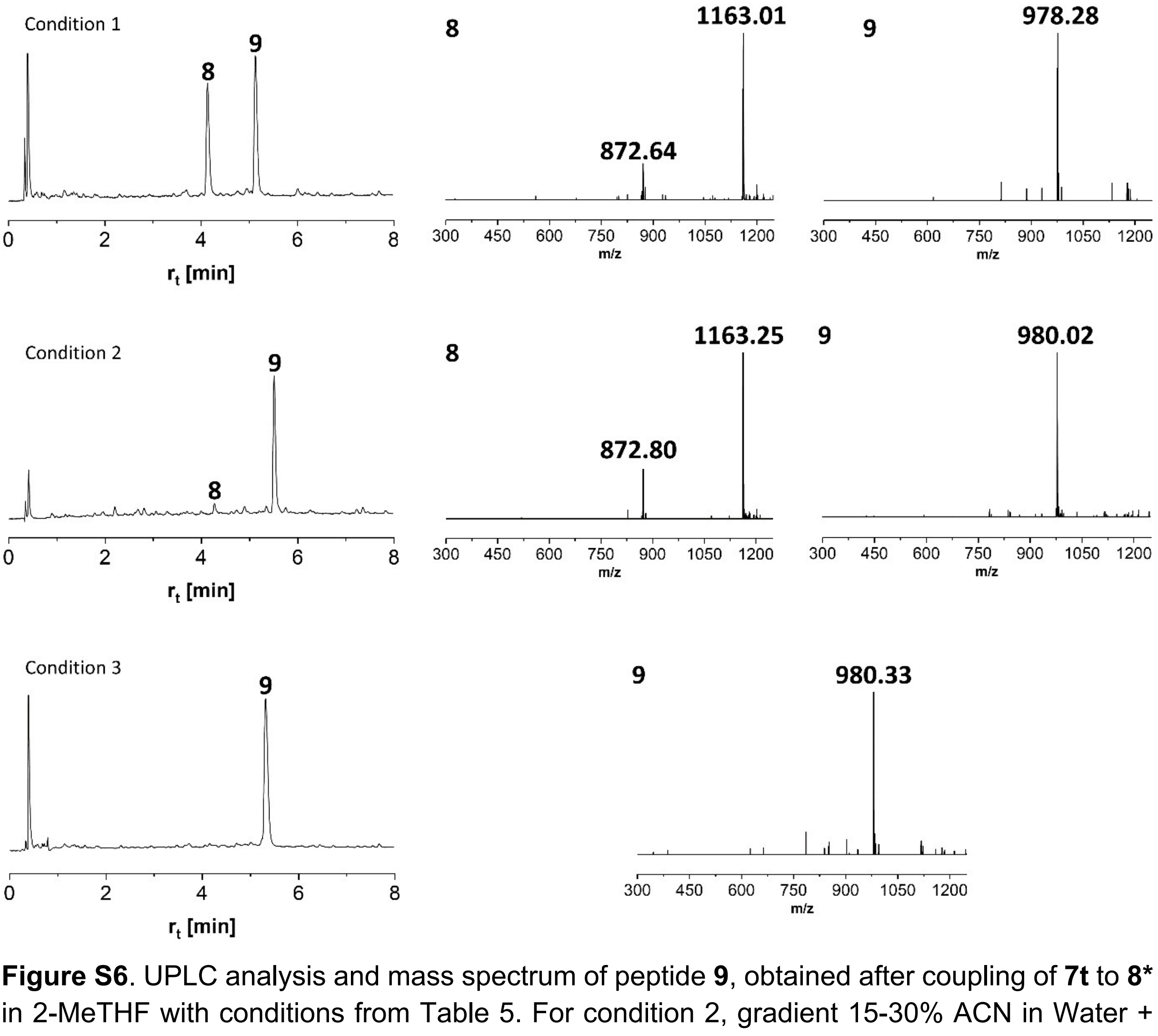

Figure S6. UPLC analysis and mass spectrum of peptide **9**, obtained after coupling of **7t** to **8*** in 2-MeTHF with conditions from Table 5. For condition 2, gradient 15-30% ACN in Water + 0.1% TFA. For others, gradient 18-30% ACN in Water + 0.1% TFA. λ = 210 nm

6. Synthesis of glycopeptides

6.1 Glycopeptide hydrazides **11** and **12**

Synthesis was performed on loaded H_2N-NH-TRT resin (for **11**: 200 mg Fmoc-Ser(tBu)-NH-NH-resin (0.11 mmol/g); for **12**: 200 mg Fmoc-Ser(αAc$_3$GalNAc)-NH-NH-resin (0.10 mmol/g)) by using the Biotage Initiator+ Alstra synthesizer. Prior to coupling of the glycoamino acid, synthesis was paused to allow for manual transfer of solutions.

Fmoc-removal: The resin was treated with 20 % piperidine in DMF for 2x5 min. The resin was washed with 3x 2 mL of DMF, each washing was performed for 1 minute.

Coupling of usual amino acid: Fmoc-protected amino acids (5 eq.) were activated with HATU/Oxyma (5 eq. each) and transferred to the resin with DiPEA (15 eq.). The final concentration of amino acid was 167 mM. Coupling was perfomed for 30 min. *Amino acids in sequence right after glycoamino acids were coupled twice.* The resin was washed with 2x 2 mL of DMF, each washing was performed for 1 minute.

Coupling of glycoamino acid: The resin was washed extensively with CH_2Cl_2. Fmoc-protected glycoamino acid, DIC and Oxyma (each 1.5 eq.) were dissolved in 2-MeTHF to 50 mM concentration. The solution was transferred to the resin. Coupling was performed for 10 min. The resin was washed with 2x 2 mL of DMF, each washing was performed for 1 minute.

Capping: see 5.1.

Removal of *O*-acetyl groups: The resin was treated with a mixture of N_2H_4 aq. solution (51% of N_2H_4)/DMF (1:1, v/v) for 30 minutes. Liquid was removed and resin was washed five times with DMF and five times with CH_2Cl_2.

TFA cleavage and work-up: see 5.1.

6.1.1 Ac-(AP**gT**TS**gT**TS)$_5$-NH-NH$_2$ (**11**)

The glycopeptide was assembled on 200 mg Fmoc-Ser(*t*Bu)-NH-NH-resin (0.11 mmol/g). Preparative HPLC 07-17% B in 40 min. Yield: 21.95 mg (3.76 µmol), 17%. UPLC-MS: t_R = 3.21 min 07-17% B in 10 min); m/z = 1947.4, ($C_{232}H_{389}N_{52}O_{121}$ $(M+3H)^{3+}$, calcd.: 1947.6), 1460.8, ($C_{232}H_{390}N_{52}O_{121}$ $(M+4H)^{4+}$, calcd.: 1461.0), 1169.0, ($C_{232}H_{391}N_{52}O_{121}$ $(M+5H)^{5+}$, calcd.: 1169.0).

6.1.2 Ac-(APTT**gS**TT**gS**)$_5$-NH-NH$_2$ (**12**)

The glycopeptide was assembled on 200 mg Fmoc-Ser(αAc$_3$GalNAc)-NH-NH-resin (0.10 mmol/g). Preparative HPLC 07-17% B in 40 min. Yield: 10.16 mg (1.74 µmol), 9%. UPLC-MS: t_R = 4.16 min (07-17% B in 10 min); m/z = 1947.5, ($C_{232}H_{389}N_{52}O_{121}$ $(M+3H)^{3+}$, calcd.: 1947.6), 1460.9, ($C_{232}H_{390}N_{52}O_{121}$ $(M+4H)^{4+}$, calcd.: 1461.0), 1168.8, ($C_{232}H_{391}N_{52}O_{121}$ $(M+5H)^{5+}$, calcd.: 1169.0).

6.2 Cysteine glycopeptide **13**

Synthesis was performed on 200 mg Rink Amide resin (0.20 mmol/g) by using the Biotage Initiator+ Alstra synthesizer. Prior to coupling of the glycoamino acid, synthesis was paused to allow for manual transfer of solutions. Individual steps were performed as described in 6.1.

6.2.1 CP**gT**TS**gT**TS-(AP**gT**TS**gT**TS)$_4$ (**13**)

Preparative HPLC 07-17% B in 40 min. Yield: 20.87 mg (3.59 µmol), 9%. UPLC-MS: t_R = 4.47 min (07-17% B in 10 min); m/z = 1939.0, ($C_{230}H_{386}N_{51}O_{120}S$ $(M+3H)^{3+}$, calcd.: 1939.3), 1454.6, ($C_{230}H_{387}N_{51}O_{120}S$ $(M+4H)^{4+}$, calcd.: 1454.7), 1163.9, ($C_{230}H_{388}N_{51}O_{120}S$ $(M+5H)^{5+}$, calcd.: 1164.0).

6.3 Experiments with glycopeptides containing O-acetyl-protected sugars

11

6.3.1 Loss of acetyl groups in acidic solution

Glycopeptide hydrazide **11'** (Ac-(AP**T(αAc$_3$GalNAc)**TS**T(αAc$_3$GalNAc)**TS)$_5$-NH-NH$_2$) was prepared as described in 6.1 but without removing O-acetyl groups. After TFA cleavage, glycopeptide **11'** was dissolved either in water + 0.1% TFA solution (pH 1), or in phosphate buffer at pH 7. After 3 hours, solutions were analyzed.

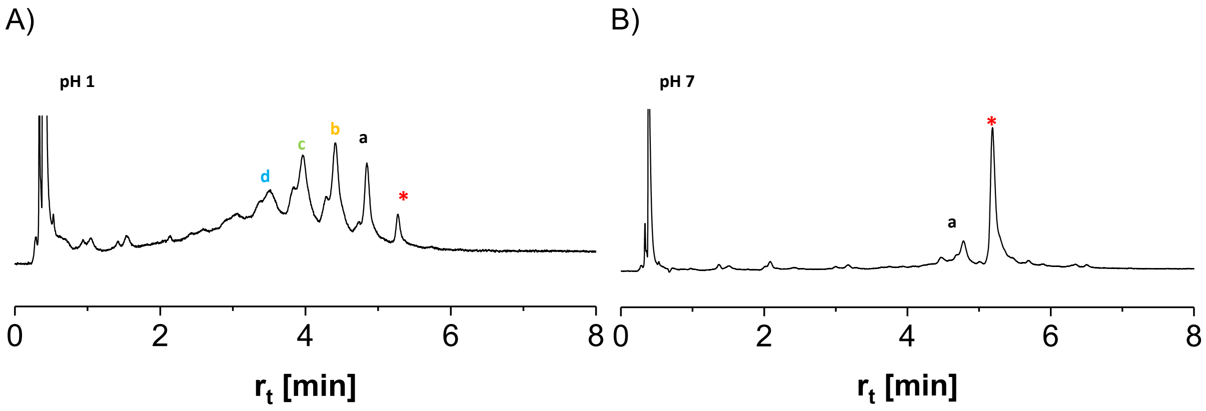

Figure S7. UPLC analysis of glycopeptide **11'** with *O*-Ac-protected sugars after 3 h incubation in aqueous soution at (A) pH 1 or (B) pH 7. Asterisk indicates targeted glycopeptide, a b c d letters indicate peptides lacking one, two, three, four acetyl groups. Gradient 25-40% ACN in Water + 0.1% TFA. λ = 210 nm

6.3.2 Synthesis of glycopeptide with O-acetyl protected sugars

After the last Fmoc-deprotection during the synthesis of peptide **12**, a small sample (~4 mg) of resin was taken. Peptide **12’** was cleaved from the small sample as described in 6.1, omitting only the treatment of resin with hydrazine hydrate:DMF solution.

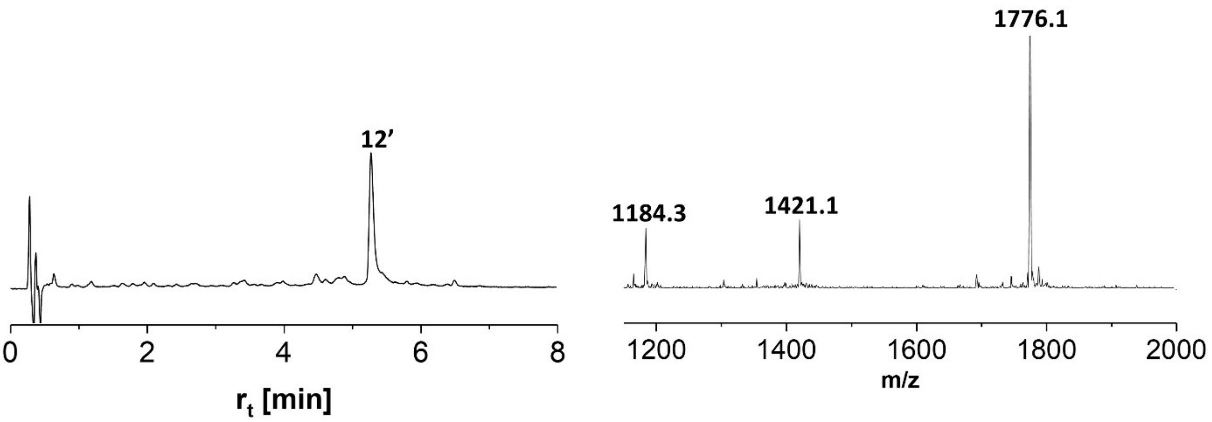

Figure S8. UPLC-MS analysis of glycopeptide **12’**. Gradient: 25-40% ACN in Water + 0.1% TFA. λ = 210 nm.

6.3.3 Cleavage of O-acetyl protecting groups from sugars in solution

An aqueous hydrazine solution (51% hydrazine,100 µL) was added to the residue obtained after ether precipitated peptide. After 30 minutes, peptide solution was analyzed with UPLC-MS.

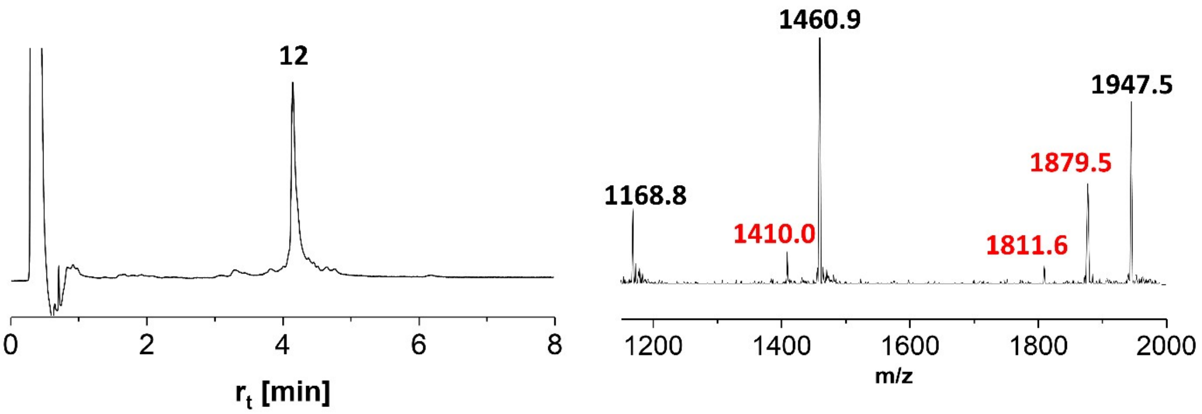

Figure S9. UPLC-MS analysis of glycopeptide **12** obtained after removal of O-acetyl groups from **12’** in solution. Gradient: 07-17% ACN in Water + 0.1% TFA. λ = 210 nm.

7. Native chemical ligation of glycopeptides

Ac-(APgTTSgTTS)$_5$-CPgTTSgTTS-(APgTTSgTTS)$_4$-NH$_2$ (S14)

Glycopeptide hydrazide **11** (2.78 mg, 0.48 μmol) was dissolved in 96 μL of ligation buffer A (6M Gn-HCl, pH = 3.1) to reach a concentration of 5 mM. MPAA (3.3 mg) was added to this solution, forming a saturated MPAA emulsion at ~200 mM concentration. A solution (0.74 μL) of acetylacetone in water (10%) was added (1.5 eq., 0.72 μmol). The mixture was vortexed. Aliquots were withdrawn after 90 min and 150 min. Formation of thioester **11TE** was completed after 150 min.

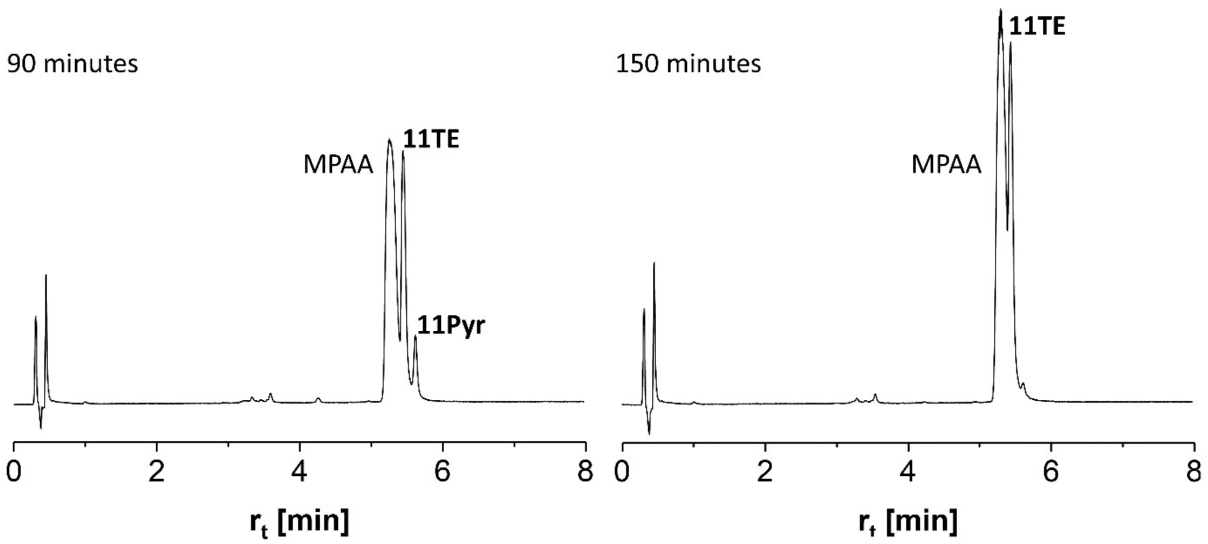

Figure S10. UPLC analysis of thioesterification of **11** after 90 min and 150 min. At 90 minutes, starting glycopeptide **11** is consumed, but complete conversion to **11TE** is not yet achieved because the peptide-pyrazolate intermediate **11Pyr** is still present. After 150 minutes, formation of peptide thioester **11TE** is completed.

Glycopeptide **13** (2.49 mg, 0.42 μmol) was dissolved in 84 μL of ligation buffer B (6M Gn-HCl, 0.2M Na_2HPO_4, pH = 8.50) to reach a concentration of 5 mM. TCEP hydrochloride salt (1.2 mg) was added to reach a concentration of 50 mM. This mixture was combined with the mixture of **11TE**. The pH was adjusted to ~7.1 with 1M NaOH solution. After vigorous vortexing, the reaction was allowed to proceed at ambient temperature. Aliquots were withdrawn at indicated time points for UPLC analyses. After completion of reaction, the mixture was forwarded to one-pot desulfurization (chapter 8).

Ac-(APTTgSTTgS)$_5$-CPgTTSgTTS-(APgTTSgTTS)$_4$-NH$_2$ (S15)

Glycopeptide hydrazide **12** (2.92 mg, 0.50 μmol) was dissolved in 100 μL of ligation buffer A (6M Gn-HCl, pH = 3.09) to reach a concentration of 5 mM. MPAA (3.4 mg) was added to this solution, forming a saturated MPAA emulsion of ~200 mM concentration. A solution (0.77 μL) of acetylacetone in water (10%) was added (1.5 eq., 0.75 μmol). The mixture was vortexed. An aliquot was withdrawn after 60 min and formation of thioester **12TE** was completed.

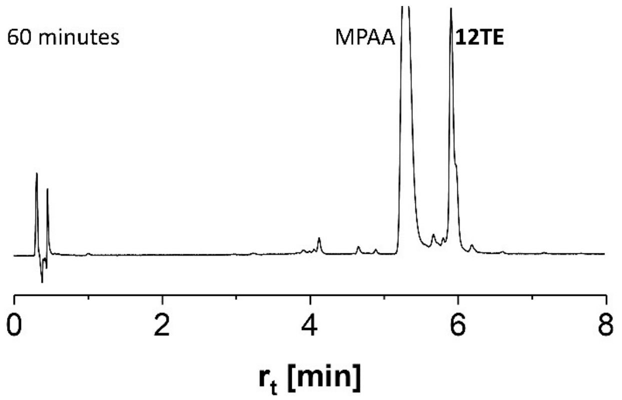

Figure S11. UPLC analysis of thioesterification of **12.**

Glycopeptide **13** (2.49 mg, 0.42 µmol) was dissolved in 84 µL of ligation buffer B (6M Gn-HCl, 0.2M Na_2HPO_4, pH = 8.50) to reach a concentration of 5 mM. TCEP hydrochloride salt (1.2 mg) was added to reach a concentration of 50 mM. This mixture was combined with the solution of **12TE**. The pH was adjusted to ~7.1 with 1M NaOH solution. After vigorous vortexing, the reaction was allowed to proceed at ambient temperature. Aliquots were withdrawn at indicated time points for UPLC analyses. After completion of reaction, the mixture was forwarded to one-pot desulfurization (chapter 8).

8. One-pot add-and-done desulfurization of glycopeptides

Add-and-Done desulfurization was performed by adopting a previously described protocol.[6]

A stock solution (6.66 M) of $NaBEt_4$ (1 gram/mL) in Milli-Q water was freshly prepared. The pH of the ligation mixture was lowered to 3 with 5M HCl. EtOAc (500 μL) was added to each Eppendorf tube. Mixtures were vortexed for 30 seconds, then centrifuged for 30 seconds. Organic layers were discarded, and the extraction procedure was repeated twice. UPLC analysis confirmed the absence of MPAA in ligation mixtures. The aqueous solutions were diluted with 400 μL of desulfurization buffer (6M Gn-HCl, 0.2M monosodium citrate, pH = 4.52). TCEP hydrochloride salt (5.75 mg) was added to reach 200 mM concentration. The pH value was adjusted to 4.5. From the $NaBEt_4$ stock solution 1.5 μL were withdrawn and added to reach a concentration of 100 mM. Gas evolution was immediately observed. The mixture was vortexed for 1 minute and submitted to purification by preparative HPLC (07-17% B in 40 min).

8.1 Ac-(AP**gT**TS**gT**TS)$_{10}$-NH_2 (**14**)

Preparative HPLC 07-17% B in 40 min. Yield: 2.66 mg (0.23 μmol), 55%. UPLC: t_R = 4.69 min (07-17% B in 10 min). ESI-HRMS (pos. mode): m/z = 1931.70 ($^{12}C_{462}H_{771}N_{107}O_{241}$ $(M+6H)^{6+}$, calcd.: 1931.69), 1931.86 ($^{12}C_{461}{}^{13}C_1H_{771}N_{107}O_{241}$ $(M+6H)^{6+}$, calcd.: 1931.85), 1932.04 ($^{12}C_{460}{}^{13}C_2H_{771}N_{107}O_{241}$ $(M+6H)^{6+}$, calcd.: 1932.02), 1932.20 ($^{12}C_{459}{}^{13}C_3H_{771}N_{107}O_{241}$ $(M+6H)^{6+}$, calcd.: 1932.19), 1932.36 ($^{12}C_{458}{}^{13}C_4H_{771}N_{107}O_{241}$ $(M+6H)^{6+}$, calcd.: 1932.35), 1932.54 ($^{12}C_{457}{}^{13}C_5H_{771}N_{107}O_{241}$ $(M+6H)^{6+}$, calcd.: 1932.52).

8.2 Ac-(APTT**gS**TT**gS**)$_5$-(AP**gT**TS**gT**TS)$_5$-NH_2 (**15**)

Preparative HPLC 07-17% B in 40 min. Yield: 1.74 mg (0.15 μmol), 36%. UPLC: t_R = 4.87 min (07-17% B in 10 min). ESI-HRMS (pos. mode): m/z = 1931.71 ($^{12}C_{462}H_{771}N_{107}O_{241}$ $(M+6H)^{6+}$, calcd.: 1931.69), 1931.86 ($^{12}C_{461}{}^{13}C_1H_{771}N_{107}O_{241}$ $(M+6H)^{6+}$, calcd.: 1931.85), 1932.02 ($^{12}C_{460}{}^{13}C_2H_{771}N_{107}O_{241}$ $(M+6H)^{6+}$, calcd.: 1932.02), 1932.20 ($^{12}C_{459}{}^{13}C_3H_{771}N_{107}O_{241}$ $(M+6H)^{6+}$, calcd.: 1932.19), 1932.36 ($^{12}C_{458}{}^{13}C_4H_{771}N_{107}O_{241}$ $(M+6H)^{6+}$, calcd.: 1932.35), 1932.52 ($^{12}C_{457}{}^{13}C_5H_{771}N_{107}O_{241}$ $(M+6H)^{6+}$, calcd.: 1932.52).

9. References

1 C. Qin, Z. Liu, M. Ding, J. Cai, J. Fu, J. Hu, P. H. Seeberger and J. Yin, *J. Carbohydr. Chem.*, 2020, **39**, 374.

2 A. K. Balcerzak, S. S. Ferreira, J. F. Trant and R. N. Ben, *Bioorg. Med. Chem. Lett.*, 2012, **22**, 1719.

3 S. Yamamura, M. Toda and Y. Hirata, *Org. Synth.*, 1973, **53**, 86.

4 Z. Wu, X. Guo and Z. Guo, *Chem. Comm.*, 2010, **46**, 5773.

5 D. Boeglin and W. D. Lubell, *J. Comb. Chem.*, 2005, **7**, 864.

6 Z. Sun, W. Ma, Y. Cao, T. Wei, X. Mo, H. Y. Chow, Y. Tan, C. H. P. Cheung, J. Liu, H. K. Lee, E. C. M. Tse, H. Liu and X. Li, *Chem*, 2022, **8**, 2542.
